# Abundance of the Quorum-Sensing Factor Ax21 in Four Strains of *Stenotrophomonas maltophilia* Correlates with Mortality Rate in a New Zebrafish Model of Infection

**DOI:** 10.1371/journal.pone.0067207

**Published:** 2013-06-26

**Authors:** Mario Ferrer-Navarro, Raquel Planell, Daniel Yero, Elías Mongiardini, Gerard Torrent, Pol Huedo, Paula Martínez, Nerea Roher, Simon Mackenzie, Isidre Gibert, Xavier Daura

**Affiliations:** 1 Institut de Biotecnologia i de Biomedicina (IBB), Universitat Autònoma de Barcelona (UAB), Cerdanyola del Vallès, Barcelona, Spain; 2 Departament de Genètica i de Microbiologia, Universitat Autònoma de Barcelona (UAB), Cerdanyola del Vallès, Barcelona, Spain; 3 Institute of Aquaculture, University of Stirling, Stirling, Scotland, United Kingdom; 4 Catalan Institution for Research and Advanced Studies (ICREA), Barcelona, Spain; University of Malaya, Malaysia

## Abstract

*Stenotrophomonas maltophilia* is a Gram-negative pathogen with emerging nosocomial incidence. Little is known about its pathogenesis and the genomic diversity exhibited by clinical isolates complicates the study of pathogenicity and virulence factors. Here, we present a strategy to identify such factors in new clinical isolates of *S. maltophilia*, incorporating an adult-zebrafish model of *S. maltophilia* infection to evaluate relative virulence coupled to 2D difference gel electrophoresis to explore underlying differences in protein expression. In this study we report upon three recent clinical isolates and use the collection strain ATCC13637 as a reference. The adult-zebrafish model shows discrimination capacity, i.e. from very low to very high mortality rates, with clinical symptoms very similar to those observed in natural *S. maltophilia* infections in fish. Strain virulence correlates with resistance to human serum, in agreement with previous studies in mouse and rat and therefore supporting zebrafish as a replacement model. Despite its clinical origin, the collection strain ATCC13637 showed obvious signs of attenuation in zebrafish, with null mortality. Multilocus-sequence-typing analysis revealed that the most virulent strains, UV74 and M30, exhibit the strongest genetic similitude. Differential proteomic analysis led to the identification of 38 proteins with significantly different abundance in the three clinical strains relative to the reference strain. Orthologs of several of these proteins have been already reported to have a role in pathogenesis, virulence or resistance mechanisms thus supporting our strategy. Proof of concept is further provided by protein Ax21, whose abundance is shown here to be directly proportional to mortality in the zebrafish infection model. Indeed, recent studies have demonstrated that this protein is a quorum-sensing-related virulence factor.

## Introduction

*Stenotrophomonas maltophilia* is a non-fermentative Gram-negative bacterium with increasing incidence in hospital environments [1], [2]. This obligate aerobic bacterium can be found in almost any aquatic or humid environment, including drinking-water supplies [3] and is now recognized as an emerging nosocomial pathogen. *S. maltophilia* has been associated with respiratory infections, septicemia, biliary sepsis, endocarditis, conjunctivitis, meningitis, urinary tract infections and various wound infections in immunocompromised patients as well as in cystic fibrosis (CF) patients [2], [4], [5]. Currently, *S. maltophilia* has been isolated from the lungs of approximately 10% of the CF patients in USA and up to 25% of those in Europe [1] and displays significant morbidity and mortality rates among debilitated patients [2], [5], [6], [7], [8].

*S. maltophilia* exhibits high-level intrinsic resistance to a variety of structurally unrelated antibiotics, including β-lactams, quinolones, aminoglycosides, tetracycline, disinfectants and heavy metals [9], [10]. Intrinsic resistance may be due to reduced outer-membrane permeability, changes in LPS structure, the production of multidrug efflux pumps and the presence of integrons for site-specific insertion of resistance gene cassettes [11], [12]. The production of melanin-like pigments and biofilms have also been linked to antimicrobial resistance [12]. Thus, the adhesion of *S. maltophilia* to medical implants, catheters and epithelial cells, leading to the formation of biofilms, confers natural protection against different antimicrobial agents and host immune defenses. In this regard, the development of therapies against *S. maltophilia* infection represents a significant challenge for both clinicians and microbiologists. In addition, knowledge of virulence factors is scarce and limited to homology relationships.

Recently, four *S. maltophilia* genomes have been fully sequenced and assembled (strains K279a, R551–3, JV3 and D457), and putative virulence factors have been identified by homology relationships [11], [13], [14]. These factors include type I, II, IV, and V protein-secretion systems, various pili, fimbriae, putative adhesins, tissue-degradative exoenzymes, siderophores, quorum-sensing factors and proteins involved in polysaccharide synthesis and intracellular signaling. Some fimbrial structures have been identified and characterized and their role in adhesion to epithelial cells and abiotic surfaces has been demonstrated [15]. However, the level of understanding of this bacterium's pathogenicity and virulence is still limited and the number of *S. maltophilia* strains phenotypically and genotypically analyzed is minor. Furthermore, there is considerable uncertainty about the route(s) of infection of *S. maltophilia*. Additionally, the remarkable diversity of sources from which *S. maltophilia* strains have been isolated indicates that these bacteria exhibit a high level of genomic plasticity and metabolic heterogeneity, possibly allowing them to expand their pathogenic potential. Heterogeneity is also illustrated among *S. maltophilia* isolates recovered from a single patient, showing phenotypic variation over time as a consequence of horizontal gene transfer or high mutation rates [16].

In order to provide answers to some of the above the development of an appropriate infection models is essential. Previous studies suggest a limited invasiveness of *S. maltophilia* in mice, as indicated by a transient and minimal presence of the microorganism in animal organs. For example, *S. maltophilia* CF strains were shown to cause no mortality in a neonatal mouse model of respiratory tract infection [17]. Despite this lack of robust invasiveness, mouse models of *S. maltophilia* infection have provided information on the type of host immune response induced by this opportunistic pathogen [2]. More recently, a model of acute respiratory infection in DBA/2 mice following a single exposure to aerosolized bacteria enabled the investigation of bacterial clearance, histological damage, and inflammatory response in the lungs of infected mice [18]. However, while bacterial colonization and mortality were achieved in that model, infection disseminated at a very low rate even using high doses of a virulent strain and most of the animals were able to resolve *S. maltophilia* lung colonization in a relatively short time period. For that reason, animal-weight loss is often taken as the best criterion for the comparison of pathogenesis and virulence of tested strains [18], [19]. In addition, lung infection models tend to be time-consuming, labor intensive and have associated welfare issues. Therefore, alternative, simple models of *S. maltophilia* infection are still needed to test the virulence of phenotypically and genotypically diverse strains.

In recent years the zebrafish (*Danio rerio*) has emerged as an important model of vertebrate development, human disease and microbial infection [20]. Nevertheless, to our knowledge, zebrafish has not yet been reported as a model of *S. maltophilia* infection. Our choice of adult zebrafish as a plausible model was motivated by a series of observations. The existing literature in relation to other bacterial pathogen models successfully developed in the zebrafish [20], [21], [22]. *S. maltophilia* as a natural pathogen of fish causing infectious intussusception syndrome in adult channel catfish [23]. The inclusion of a wild-type phenotypic population diversity as opposed to inbred mouse lines. The adaptive and innate immune system of zebrafish has significant similarities to mammalian systems [24], [25]. The zebrafish is recognized as an important vertebrate model with genomic enablement and last, but not least relevant, ethical, economic and process-simplicity considerations.

In the study presented here, we have tested a combined approach that uses an adult-zebrafish model of *S. maltophilia* infection for the evaluation of relative virulence and the successive analysis by fluorescence-based two-dimensional Difference in-Gel Electrophoresis (DIGE) [26] of the underlying differences in protein expression, in a quest for virulence factors. This analysis was applied to three recent clinical isolates and the collection strain ATCC13637, as a reference. Although a colloidal Coomassie-stained 2DE proteomic analysis to find heat-induced changes in *S. maltophilia* protein abundance has been reported [27], to our knowledge no quantitative proteomic comparison between *S. maltophilia* strains with distinct virulence phenotypes has been previously performed.

## Results

### Zebrafish Infection Model Confirms Attenuation of Collection Strain and Points at Varying Virulence of the Clinical Isolates

**Figure 1:**
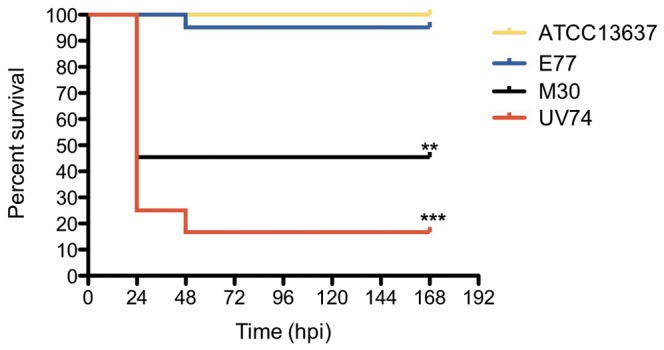
Survival curves of adult zebrafish injected with *S. maltophilia* ATCC13637, E77, M30, UV74 (10^8^ cfu in 20 µl). Control groups injected with heat-inactivated UV74 (10^8^ cfu in 20 µl) and sterile PBS (20 µl) presented no mortality (not shown). Zebrafish mortalities were recorded for 168 h post-infection (hpi) and survival curves analyzed using the Kaplan-Meier method (log-rank test: ***, *p*≤0.001; **, *p*≤0.01).

Adult zebrafish were used as an infection model to determine the virulence of the individual strains. An intraperitoneal injection of 10^8^ cfu resulted in strain-dependent mortality rates (Figure 1), where UV74 was the most aggressive, with 84% mortality 48 h post-injection (p.i.), followed by strains M30 (55%), E77 (5%) and ATCC13637 (0%). Although the experimental period was during 7 days, in all cases mortality occurred during the first 48 h. Clinical symptoms of dead fish included cutaneous hemorrhage under the lower jaw, on the belly, and around the anus; all dead fish had a distended abdomen containing bloody or clear fluid and severe enteritis with intussusception in the lower intestine. All isolates obtained from post-mortem UV74 infected zebrafish were identified as *S. maltophilia* based on morphology, API (analytical profile index) and 16S rDNA sequence (data not shown). No zebrafish died when injected with 20 µl of sterile PBS (data not shown). Interestingly, injection of 10^8^ heat-inactivated UV74 bacteria did not produce mortality in zebrafish. This suggests that viable bacteria producing thermo-labile proteins are required for infection and that mortality is not caused by non-specific activation due to bacterial components such as lipopolysaccharides.

### Strain-genotyping Analysis Reveals a Distant Genetic Relationship between the Strains

**Figure 2:**
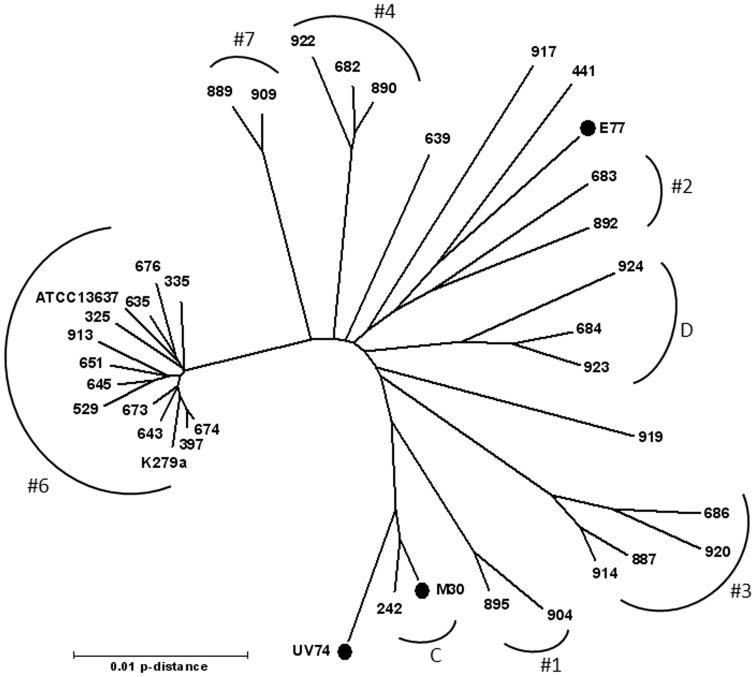
Neighbor-joining radiation tree based on the concatenated sequences of the MLST loci, showing relationships among the three recent clinical isolates (solid circles), the ATCC13637 strain and 35 previously characterized *S. maltophilia* pathogenic strains [28]. Previously defined genomic groups [28], [29] are indicated (#1 to #7, C and D).

The genetic relationship of the four *S. maltophilia* strains was assessed using multilocus sequence typing (MLST). The isolates were compared and organized based on similarities among seven housekeeping genes (*atpD*, *gapA*, *guaA*, *mutM*, *nuoD*, *ppsA* and *recA*). This analysis resulted in the three clinical strains being classified into new, different sequence types (M30 as ST-76, UV74 as ST-77 and E77 as ST-81), indicating they are not clonally related despite being isolated from a common hospital setting. Nevertheless, strains M30 and UV74 share the same allele for genes *atpD* and *mutM*. The collection strain ATCC13637 had been already characterized by MLST and assigned the ST-14 [28]. In addition, thirty-five concatenate sequences from different *S. maltophilia* strains, isolated from human clinical cases, were obtained from public databases and used to provide the context for the genotypic classification of the three clinical isolates and the collection strain. Strains were chosen such that they cover as far as possible the full genetic breadth of the species [28]. The phylogenetic analysis revealed distant genetic relationships between the three clinical strains and the reference that according to a previous classification based on AFLP fingerprinting [29] would belong to a different genomic group (Figure 2). Strains M30 and UV74 show the closest genetic relationship, clustering within genomic group C as previously described by Kaiser *et al.* [28]. The MLST results imply that the three pair wise (collection:clinical) proteomic analyses described below are non-redundant.

### The Most Virulent Strain, UV74, also shows the Most Resistant Phenotype

MIC determination included the following antibiotic families: tetracyclines, aminoglycosides, macrolides, sulfonamides, chloramphenicol and fluoroquinolones (Table 1). UV74 presented the most resistant phenotype to the antibiotics tested. The most distinguishing feature of this strain is its significantly higher MIC for ciprofloxacin (16 to 64-fold). In addition, the new clinical isolates were significantly more resistant to kanamycin than the ATCC13637 strain (8 to 32-fold).

**Table 1:** MICs for several antibiotics against the *S. maltophilia* collection and clinical strains.

	MIC (µg/ml)										
Strain	Tc	Mino	Gent	Kan	Trim	Sul	Cipro	Nor	Levo	Ery	Cm
ATCC13637	12.8	0.2	3.2	16.0	16.0	512.0	0.2	12.8	0.8	6.4	6.4
M30	12.8	0.1	3.2	128.0	4.0	256.0	0.8	2.0	0.4	12.8	6.4
UV74	25.6	0.8	3.2	512.0	32.0	512.0	12.8	12.8	0.8	12.8	12.8
E77	6.4	0.2	3,2	256.0	8.0	64.0	0.4	12.8	0.2	12.8	6.4

Tc, tetracycline; Mino, minocycline; Gent, gentamicin; Kan, kanamycin; Trim, trimethoprim; Sul, sulphamethoxazole; Cipro, ciprofloxacin; Nor, norfloxacine; Levo, levofloxacin; Ery, erythromycin; Cm, chloramphenicol.

### The Collection Strain shows Reduced Biofilm-formation and Swimming Capacities

The strains presented similar growth curves except for E77, whose doubling time during the exponential phase was 1.3 h as opposed to 1 h for the other three strains (Figure S1). The capacity to form biofilms on polystyrene plates was tested (Table 2) and statistical analysis revealed that the collection strain had a reduced ability to form biofilms in comparison to the clinical strains. M30 presented the greatest biofilm-formation capacity.

**Table 2:** Swimming, twitching and biofilm formation of the *S. maltophilia* collection and clinical strains.

	Swimming^a^ (cm)	Twitching^a^ (cm)	Biofilm formation^a^ (OD_620nm_)
Strain	24 h	24 h	24 h
ATCC13637	2.1±0.5	1.5±0.2	0.16±0.02
M30	3.1±0.5^b^	1.4±0.2	0.33±0.02^b^
UV74	3.8±0.5^b^	1.4±0.2	0.31±0.02^b^
E77	3.4±0.5^b^	1.6±0.2	0.19±0.02^b^

a Values represent the mean and standard deviation.

b *p*≤0.001 significance of difference with ATCC13637 by one-way analysis of variance (ANOVA) with a Bonferroni’s multiple comparison post-test.

The analysis of different types of bacterial motility (swimming, twitching, swarming) showed that ATCC13637 displays significantly reduced swimming compared to the clinical strains (Table 2). Twitching migration capabilities however were similar among the four strains. Finally, all strains presented swarming motility consisting of branches or tentacles radiating from the inoculation point. Although E77 presented swarming under the conditions described in materials and methods, observation of this type of motility in the other strains required 12 days of incubation at 30°C in the medium described by Kholer *et al*. [30]. To our knowledge, this is the first time swarming has been described for *S. maltophilia*.

### Serum Sensitivity Correlates with Virulence in Zebrafish

**Figure 3:**
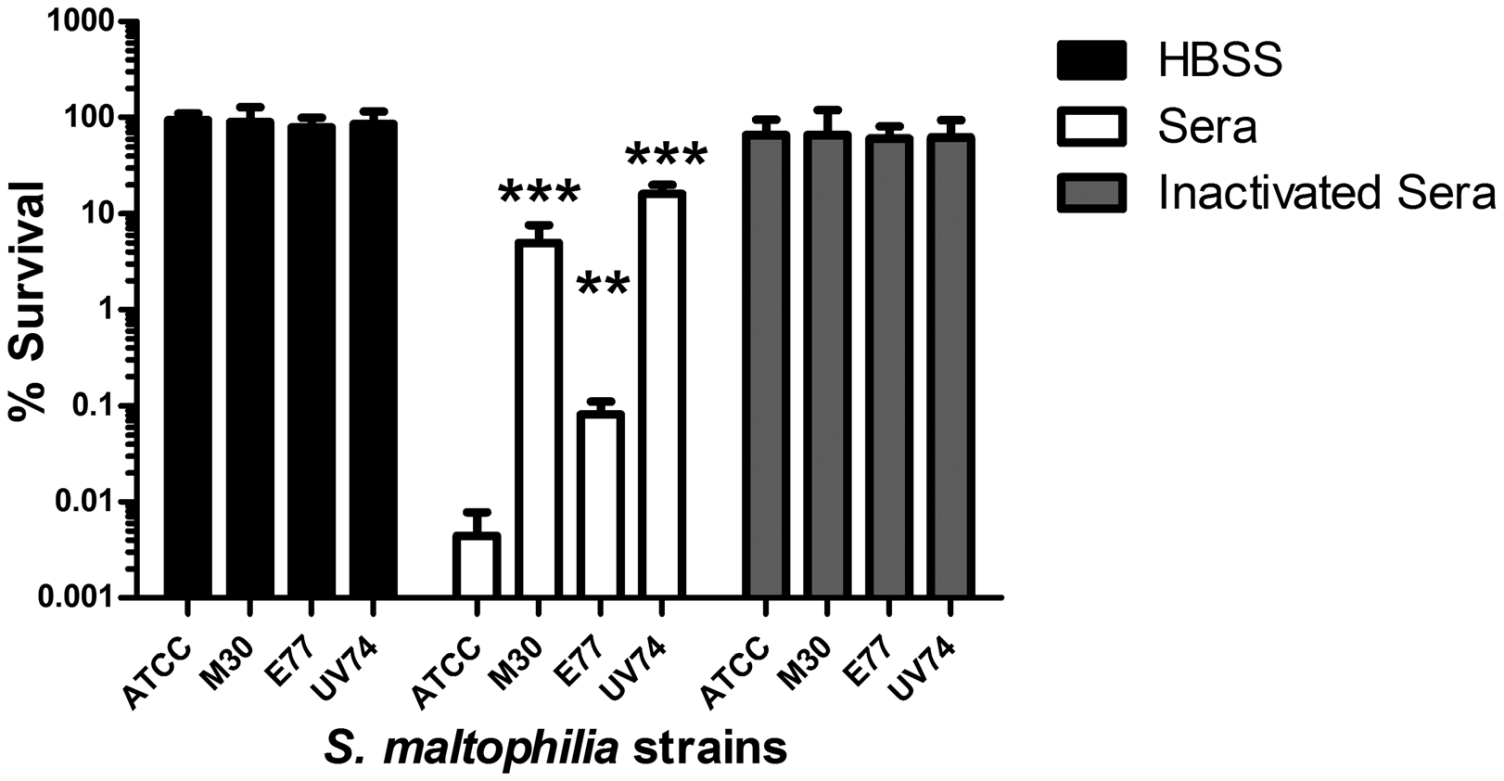
*S. maltophilia* serum-sensitivity assay. Percentage of surviving cells after 90 minutes of incubation in the presence of Hank’s balanced salt solution, human serum or inactivated serum. The values represent the mean of three replicas of the three independent experiments. **, *p*≤0.01; ***, *p*≤0.0001 significance of difference with ATCC13637 by unpaired *t*-test with Welch correction for unequal variances.

Resistance of the four strains to antibody/complement-mediated bactericidal action of serum was also tested (Figure 3). The ATCC13637 and E77 strains clearly showed a higher sensitivity to the bactericidal action of serum (0.0044% and 0.082% survival, respectively) compared to the M30 and UV74 (5% and 16% survival, respectively). As a control, when the incubation was performed with Hank’s balanced salt solution (HBSS) or inactivated serum no mortality was observed. Notably, these results correlate with zebrafish mortality (*p* = 0.059).

### Adhesion to HeLa Cells Correlates with Serum Sensitivity

**Figure 4:**
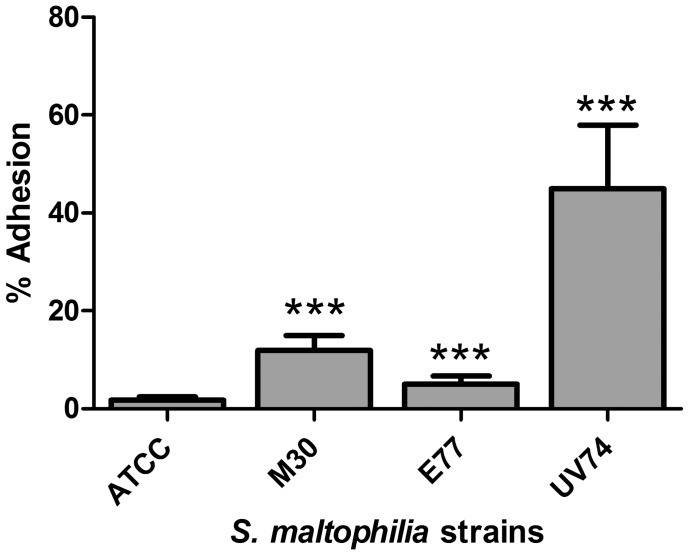
*S. maltophilia* adhesion to HeLa cells. Percentage of bacteria adhered to HeLa cells after 120 min of incubation in 24-well plates. The data correspond to the mean and standard deviation of three different assays carried out in triplicate. ***, *p*≤0.001 significance of difference with ATCC13637 by unpaired *t*-test with Welch correction for unequal variances.

To test the relative adhesion capacities of the *S. maltophilia* strains to eukaryotic cells, adhesion experiments with HeLa cells were performed (Figure 4). For ATCC13637, the number of adhered bacteria to HeLa cells after 2 h of incubation was 1.7% of the initial bacterial load. This was significantly lower than the percentages found for M30 (12%), E77 (5%) and UV74 (45%). This results correlate with serum sensitivity (*p* = 0.008) and also follow the strain-virulence order.

### Differential Proteomics Points at Specific Processes and Highlights a Protein that Correlates with Virulence

To analyze potential differences in the protein profiles of the collection and clinical strains, independent samples were taken from each strain in the exponential-growth phase (OD = 1) and were Cy-dye labeled and pairwise separated by 2DE. An example of the resulting fluorescence images is shown in Figure S2, with the ATCC13637 sample in red and the clinical sample in green. A total of 1807, 1776 and 1677 protein spots were detected in the ATCC13637-M30, ATCC13637-E77 and ATCC13637-UV74 gels, respectively. Using a 1.5-fold threshold for clinical:collection abundance ratio, ca. 100 spots displayed significant differential content of protein from collection and clinical sources (*p*<0.05, ANOVA test). These spots were excised and in-gel digested with trypsin. Following MALDI-MS time-of-flight analysis, 38 proteins were identified for which the absolute value of the ratio was ≥1.5 for at least two clinical isolates. The low identification rate (38 out of 100 differential spots) stems partly from the fact that the identification is performed on the analytical gels, to avoid uncertainties with mismatching spots on the preparative silver-stained gels. The protein amount loaded on the analytical gels is low (25 µg per sample), making the identification of weak-intensity spots impossible. Some of the proteins were identified in several spots, indicating the presence of different post-translationally modified forms. A detailed list of the identified proteins and quantified abundance differences is given in Table S1, with the corresponding statistical analysis provided in Table S2. Example fluorescence images and expression profiles of some of the identified proteins are shown in Figures S2 and S3, respectively. In addition, a survey on orthologs of the 38 proteins that have been linked to pathogenesis, virulence or resistance has been performed and summarized in Table S3.

Among the proteins with altered abundance in clinical strains there are enzymes involved in the biosynthesis of fatty acids and other cell-wall components, enzymes involved in energy-related and other metabolic pathways, proteases, an RNA polymerase subunit, outer membrane proteins with receptor and transport activities and two completely uncharacterized proteins. The first 12 proteins (enzymes) in Table S1 are assigned to a common network by predicted pairwise interactions (see Table S3). Likewise, proteins 13 to 17 in Table S1 appear also cross connected. Indeed, some of them are known to share a role in biofilm formation and quorum sensing. Note that the ortholog information provided in Table S3 is not necessarily exportable to *S. maltophilia*. The molecular mechanisms underlying pathogenesis, virulence and resistance may vary not only between species but also between strains within a species. Furthermore, ortholog proteins involved in parallel mechanisms in two closely related species might still do so in different ways. For example, various proteins in Table S1, with known or suggested role in pathogenesis in other species appear significantly upregulated in one clinical isolate and downregulated in another, the most striking case being the LptD transporter. This protein is essential for the translocation of Lipid A across the outer membrane and, by extension, of LPS presence in the surface, and is strongly upregulated in E77 and downregulated in UV74 (remarkably the most virulent strain). While bearing this limitation in mind, comparison of Tables S1 and S3 highlights the following proteins as potentially relevant to pathogenesis, virulence or resistance: FabD, AccC, AcsA, PdhB, AcnB, enoyl-CoA hydratase, MurA, RpoA, LptD, putative TonB-dependent receptors Smlt3444 and Smlt4151, putative FadL, putative OmpA Smlt0955, PepO and putative Ax21. The latter protein, quorum-sensing factor Ax21, shows a strong correlation with the results of virulence in zebrafish challenges (*p* = 0.022) as well as with the strain order for adhesion to HeLa cells and serum sensitivity. In addition to these candidates, on the sole basis of the proteomics data (Table S1) the following proteins also deserve special attention: FadI, SucB, Bcd, OprP, putative exported peptidase S9, NuoG, UPF0234 family protein and hypothetical protein Smlt3796. Note that FadI and SucB, which are predicted to interact (Table S3), have a similar expression behaviour (Table S1), strongly downregulated in UV74 and weakly upregulated in M30 and E77.

## Discussion

The elucidation of molecular mechanisms leading to pathogenicity and virulence is essential to a better understanding of *S. maltophilia* infection and to the design of strategies to effectively combat it. The aim of the present study was to identify virulence factors in new clinical isolates of *S. maltophilia* by firstly establishing an alternative, simple animal model for the evaluation of relative virulence. This would then provide the basis for the analysis of underlying differences in protein expression, in a quest to identify virulence factors. To our knowledge this is the first time the zebrafish has been used as a model of *S. maltophilia* infection.

The present study shows that the adult-zebrafish model has a high discrimination capacity, i.e. from very low to very high mortality rates for different strains of *S. maltophilia*, and correlates with serum sensitivity. This is also known to correlate with virulence in mouse and rat models [17], [31]. Taken together, these properties point toward the zebrafish as a promising animal model for the study of *S. maltophilia* pathogenicity and virulence in humans.

Furthermore, the symptoms observed in the adult-zebrafish model after infection are very similar to those reported in a recent study on aquacultured adult channel catfish [23]. This includes ascites and enteritis, prolapse in the rectum and intussusception in the lower intestine, cutaneous hemorrhage in some cases. Affected fish were also listless, sought the water surface, became more lethargic as the disease progressed, and most died within a few days. These common symptoms suggest that the process following intraperitoneal infection is parallel to natural infection.

The collection strain ATCC13637 showed obvious signs of attenuation, with null zebrafish mortality in our experiments. This strain was isolated in 1960 from the oropharyngeal region of a patient with mouth cancer [32]. Since then, it has likely undergone a large number of *in vitro* serial passages. It is well known that the repeated *in vitro* sub-culturing of microorganisms during extended periods of time results in adaptation to the laboratory environment leading to changes in physiology, including pathogenic capacity and production of virulence factors. As a matter of fact, this property has been used in vaccine development to obtain live-attenuated strains [33]. This result points once more at the shortcomings of using standard laboratory strains in studies related to pathogenesis and virulence, even when their origin is clinical.

The three new *S. maltophilia* clinical isolates, M30, UV74 and E77, were chosen on the basis of their different clinical origin (decubitus ulcer, vascular ulcer and sputum, respectively). Despite being collected from patients of the same hospital and during the same year, the MLST analysis reported here demonstrates that they are not clonally related. Interestingly, the most virulent *S. maltophilia* strains (UV74 and M30) are the genetically most similar ones by means of MLST (Figure 2). Thus, M30 and UV74 cluster within the previously described genomic group C [28], which includes worldwide clinical strains isolated from tracheal secretion and blood cultures. Genetic and mutation-frequency analysis becomes particularly relevant when dealing with *S. maltophilia* clinical isolates, as the proportion of isolates showing high mutation frequencies (hypermutators) appears to be significantly higher for *S. maltophilia* than for other organisms [34]. However, whereas for different bacterial species the strains isolated from CF patients with chronic lung infections often show high mutation frequencies [35], [36], hypermutators have rarely been found among isolates from other types of infections [35], [37]. None of the strains tested in the present study were obtained from CF patients, for which reason mutation rates were not determined.

Phenotypic analysis of the four strains revealed that UV74 presents also the most resistant pattern to the set of antibacterials tested. In addition, the correlation found between zebrafish mortality, serum sensitivity and HeLa-cell adhesion points at the potential use of animal-free models for virulence-screening purposes. The correlation is particularly significant between serum sensitivity and HeLa-cell adhesion (*p* = 0.008), but also notable between zebrafish mortality and serum sensitivity (*p* = 0.059). As mentioned, a direct relationship between *S. maltophilia* virulence and serum sensitivity has been already described in a mouse model of respiratory tract infection [17] and a rat model of lung infection [31].

The demonstration of the attenuated character of ATCC13637 in the zebrafish infection model motivated the comparison of the proteomes of the three clinical isolates to the collection reference with the purpose of identifying potential virulence factors within the set of proteins showing a higher abundance in the clinical strains. Clearly, *S. maltophilia* will not necessarily show the same protein expression profiles under *in vitro* exponential-growth and *in vivo* infection conditions. It is very likely that a number of proteins relevant to pathogenicity and virulence will only be upregulated (or suppressed) in the latter case and will be therefore missed in this study. Nevertheless, there remains a certain possibility that proteins differentially expressed in the clinical and collection strains under *in vitro* exponential-growth conditions may also be related to pathogenicity and virulence, e.g. virulence factors that acquire a more constitutive character as result of adaptation to the clinical/infective environment.

Of the 38 proteins identified by differential proteomics, 24 are already documented for their potential implication in pathogenesis, virulence or resistance mechanisms in other bacteria (Table S3), validating the strategy presented here. Further proof of concept is provided by the identification of the putative quorum-sensing factor Ax21 (Smlt0387), whose abundance is shown to correlate with mortality in the zebrafish infection model (*p* = 0.02). It should be noted that this protein has been found in more than one spot in the gels, suggesting a different composition of isoforms between the collection and clinical isolates, being the more basic isoform the predominant in the latter. McCarthy *et al.* [38] have recently published a study in which deletion of *smlt0387* affects the transcription of genes encoding proteins involved in transcriptional regulation, antibiotic resistance and pilus assembly, and influences various phenotypes leading to reduced motility, reduced tolerance to some antibiotics, reduced biofilm formation and reduced virulence in the larval *Galleria mellonella* infection model. In addition, it has been shown that the homologous protein in *Xanthomonas oryzae* (60% identity), a microorganism closely related to *S. maltophilia*, triggers innate immunity in both plants and animals [39].

Ax21 has two conserved domains. One of them (pfam13505) is found in a wide range of outer-membrane-bound beta-barrel proteins. The other one (TIGR04273) is present in the sulfation-dependent quorum-sensing factor Ax21 protein family. This family consists of proteins closely related to Ax21 (Activator of XA21-mediated immunity), a 194-residue protein present in plant and animal pathogens [40] that is secreted by a type I secretion system (RaxABC) and appears to be sulfated at a tyrosine found in a motif LSYN in the N-terminal region. In *X. oryzae* the small protein Ax21 serves as a quorum-sensing factor, inducing density-dependent gene expression and thus regulating biofilm formation, bacterial motility and virulence. In addition, Ax21 is critical for *X. oryzae* virulence at low densities in rice leaves [40]. Contrary to the *Xanthomonas* case, Smlt0387 would apparently perform its intraspecies signalling activity in a non-sulfation-dependent manner [38]. Our results further suggest that in *S. maltophilia* the relative abundance of this protein is directly proportional to virulence. Further studies (in progress) will be needed to address the potential role of the other proteins identified in the pathogenesis, virulence or resistance of *S. maltophilia*.

## Materials and Methods

### Bacterial Strains, Media, and Growth Conditions

The three clinical *S. maltophilia* strains characterized in this work were isolated from different patients at the Hospital Municipal de Badalona (Barcelona, Spain) during the year 2009. The clinical origin of M30, E77 and UV74 strains were decubitus ulcer, sputum and vascular ulcer, respectively. Species identification was confirmed biochemically using the API NE system (bioMérieux). The collection strain ATCC13637, isolated in 1960 from the oropharyngeal region of a patient with mouth cancer [32], was also included in the experiments. *S. maltophilia* strains were routinely cultured o/n in Luria-Bertani (LB) media at 37°C and 250 rpm unless otherwise stated, and growth curves were monitored following the optical density at 550 nm (Figure S1).

### Zebrafish Infection Assay

Adult (9–12 months) wild-type zebrafish (*D. rerio*) were kept in a 12 h light:12 h dark cycle at 28°C and fed twice daily with dry feed. All fish used in infection experiments were transferred to an isolated system and acclimated for three days before infection. Adult zebrafish (n = 12/each condition) were infected by intraperitoneal injection (i. p.) [41] with 20 µl of a 5×10^9^ cfu/ml suspension of *S. maltophilia* strain ATCC13637 and the clinical isolates M30, E77 and UV74. These strains were previously grown at 28°C in blood agar plates (BioMérieux) for 20 h and collected directly from the plates with phosphate buffered saline (PBS). Two control groups were injected with PBS and with a heat-inactivated UV74 strain (incubation at 100°C for 30 min, at which time no viable bacteria were detectable), respectively. Fish were observed daily for signs of disease and mortality. All living injected fish were sacrificed after 7 days by MS-222 overdose. All experiments were repeated independently twice.

### Multilocus Sequence Typing and Phylogenetic Analysis

PCR amplification and sequencing of the seven housekeeping genes included in the MLST scheme was performed as previously described [28], [42]. The detailed MLST procedure and set of primers used were obtained from the *S. maltophilia* MLST database (http://pubmlst.org/smaltophilia/). Briefly, genomic DNA was extracted with GenElute Bacterial Genomics DNA Kit (Sigma-Aldrich). Fragments of the seven genes *atpD*, *gapA*, *guaA*, *mutM*, *nuoD*, *ppsA*, and *recA* were amplified using FastStart Taq DNA Polymerase (Roche, Diagnostics) as follows: initial denaturation at 95°C for 9 min followed by 30 cycles of denaturation at 94°C for 20 s; annealing at the appropriate temperature for 1 min; extension at 72°C for 50 s; final elongation at 72°C for 5 min. PCR products were treated with ExoSAP-IT (USB Products) and sequenced at Macrogen Inc. (Seoul, Korea) in both directions using standard conditions. The allele numbers for each locus and sequence type (ST) were determined by comparison with the available sequences at the *S. maltophilia* MLST database, where the data has been deposited. Phylogenetic relationships were established on the basis of the concatenated seven gene sequences without further corrections. Cluster analysis was performed with the Neighbour-Joining method, using uncorrected p-distance. The phylogenetic tree of *S. maltophilia* strains was constructed by use of MEGA software version 4 [43], together with 35 already analyzed pathogenic *S. maltophilia* strains [28] with sequence information available at the *S. maltophilia* MLST database.

### Determination of MICs

The susceptibility of *S. maltophilia* to the following antimicrobial agents was tested: tetracycline, minocycline, gentamicin, kanamycin, trimethoprim (from Apollo Scientific Ltd), sulfamethoxazole, norfloxacin, ciprofloxacin, erythromycin, levofloxacin (from Sigma-Aldrich), and chloramphenicol (from Roche Diagnostics). The MICs for these antibiotics were determined by microdilution test using 96-well plates by serial two-fold dilutions of each drug in 100 µl of LB. 100 µl of bacterial suspension (final OD_550nm_ = 0,005) were added and the antibiotic dilutions and the organism suspension were mixed and incubated at 37°C for 16 h before developing with resazurin (30 µl 0.01%) [44]. The MIC was defined as the lowest drug concentration that prevented bacterial growth. The microdilution assay followed the Clinical and Laboratory Standards Institute (CLSI, www.clsi.org) guidelines for antimicrobial susceptibility testing.

### HeLa Cell Adherence Assay

HeLa cells were grown for 24 h on 24-well tissue culture plates (TPP Techno Plastic Products AG) containing 2 ml of Minimum Essential Medium α (Invitrogen) with 10% (v/v) inactivated fetal bovine serum (Invitrogen) and Glutamax™ (Invitrogen) to 90–95% of confluence. Bacterial cultures of ATCC13637, M30, E77 and UV74 strains were grown o/n in LB medium at 37°C without agitation and resuspended in HeLa medium. Bacteria were added at a multiplicity of infection (MOI) of 50∶1 in triplicate to the confluent 24-well plates and were incubated at 37°C in a humidified atmosphere of 5% carbon dioxide for 120 minutes. Wells were gently washed five times with 2 ml of Dulbecco's Phosphate-Buffered Saline (Invitrogen) to remove non-adherent bacteria. The number of cell-attached bacteria was quantified by lysis with 0.1% Triton X-100 and serial dilution were plated onto LB plates. Adhesion was measured as a percentage between adhered and the initial cells.

### Serum Sensitivity Assay

Human serum sensitivity assay was performed following the protocol described by Waters *et al.* [17]. Briefly, bacteria were grown on LB medium to an OD_550nm_ of 0.5, washed in Hanks' balanced salt solution (HBSS) (Invitrogen), and incubated in HBSS, 60% serum or 60% heat-inactivated serum with agitation at 37°C with agitation for 90 min. The serum inactivation was performed at 56°C during 30 min. After incubation, bacteria were plated on LB plates after serial dilution. The survival was measured as a percentage between the surviving and initial cells.

### Biofilm Formation on Polystyrene Plates

Quantification of *S. maltophilia* biofilm formation was assessed by crystal violet (CV) staining in 96-well polystyrene plates. Bacterial cultures of the four different *S. maltophilia* strains were grown o/n at 37°C. Absorbance at 550 nm was adjusted to 0.1 and 200 µl were grown in a 96-well plate during 24 h at 30°C. Cells were washed three times with water, fixed at 60°C for 1 h and stained during 15 min with 200 µl of 0.1% CV. The dye was discarded, and the plate was rinsed in standing water and allowed to dry for 30 min at 37°C. Stained biofilms were exposed to 250 µl of 95% ethanol for 15 min, and the OD of the extracted dye was measured at 620 nm.

### Motility Assays

Overnight cultures of the different *S. maltophilia* strains were grown (plate or liquid medium) in LB under standard conditions. The swimming motility was determined in TrA plates (1% tryptone, 0.5% NaCl, 0.25% agar) [45]. Thus, 5 µl of adjusted o/n cultures of the different *S. maltophilia* strains were spotted on TrA plates. The twitching and swarming motility plates were LB plates at 1% agar and BM2 at 0.5% agar [46], respectively. Twitching was assessed via subagar stab inoculations (stab assay) from o/n fresh plates as previously described [47]. The twitching zones were then visualized by staining with 1% (wt/vol) crystal violet and their diameters measured. Noble agar (Difco™) was used in the preparation of the three motility assay plates. The growth halos were measured in cm after 24 h of incubation at 30°C for the swimming and twitching motilities and after 7 days at 30°C for swarming.

### Statistics

Statistical analyses were performed using the GraphPad Prism program version 5.00. Comparison of strain phenotypic data was performed by one-way analysis of variance (ANOVA) with a Bonferroni’s multiple comparison post-test or unpaired *t*-test with Welch correction for unequal variances, as indicated in figure and table captions. Survival curves of zebrafish infection experiments were analyzed using the Kaplan-Meier method. Differences were evaluated using the log-rank test. The relationship between relative protein abundance in the different strains and pathogenicity variables, such as zebrafish mortality rates and adhesion to human cells, was evaluated with Pearson’s chi-squared test. *p*≤0.05 was considered significant.

### Sample Preparation for Two-dimensional Gel Electrophoresis

20 ml of exponential culture (OD = 1) were washed with PBS 1X three times and resuspended in lysis solution (8 M urea, 2 M thiourea, 2.5% 3-[(3-cholamidopropyl) dimethylammonio]-1 propanesulfonate (CHAPS), 2% ASB-14, 40 mM Tris-HCl, pH 8.8). ASB-14 was used to increase the presence of membrane proteins in the 2DE [48]. Then, samples were disrupted by sonication and centrifuged in order to discard any insoluble cellular debris. In order to remove salts and other contaminants, samples were cleaned with 2D Clean-Up Kit (GE Healthcare). Resulting pellets were resuspended in the above-mentioned lysis solution. Protein concentration was determined with 2D-Quant Kit (GE Healthcare) and adjusted to 2 mg/ml by the addition of a DIGE labeling buffer (7 M urea, 2 M thiourea, 2.5% w/v CHAPS, 40 mM Tris, pH 8.8). A pool consisting of equal amounts of each of the two samples analyzed in the experiment was prepared as an internal standard for quantitative comparisons [49]. The clinical isolates were labeled with Cy3 and the collection strain ATCC13637 was labeled with Cy5. A third fluorescent dye, Cy2, was used to label the internal standard sample. Labeling was carried out by the addition of 400 pmol of the required Cydye in 1 µl of anhydrous N,N-dimethylformamide per 50 µg of protein. After 30 min of incubation on ice in the dark, the reaction was quenched with 10 mM lysine and the samples incubated for a further 10 min. Samples were combined according to the experimental design, using 50 µg of protein per Cy dye per gel, and diluted two-fold with isoelectric focusing (IEF) sample buffer (7 M urea, 2 M thiourea, 4% w/v CHAPS, 2% dithiothreitol [DTT], 2% pharmalytes, pH 3–10). One clinical strain and the ATCC13637 strain sample, together with an aliquot of the internal standard pool, were then separated by two-dimensional electrophoresis (2-DE) in each of the gels. This experimental design allows the accurate quantification and statistical assessment of the differences in protein abundances observed between the two sample groups.

### 2D Difference Gel Electrophoresis

The 2-DE was performed using GE Healthcare reagents and equipment. First-dimension isoelectric focusing was performed on immobilized pH gradient strips (24 cm, pH 3–10) using an Ettan IPGphor System (GE Healthcare). Samples were applied near the basic end of the strips by cup-loading, after being incubated o/n in 450 µl of rehydration buffer (7 M urea, 2 M thiourea, 2.5% w/v CHAPS, 2% ASB-14 w/v, 0.5% pharmalytes, pH 3–10, 100 mM DeStreak reagent). After focusing at 70 kVh, strips were equilibrated, first for 15 min in 10 ml of reducing solution (6 M urea, 100 mM Tris-HCl, pH 8, 30% v/v glycerol, 2% w/v SDS, 5 mg/ml dithiothreitol [DTT]) and then in 10 ml of alkylating solution (6 M urea, 100 mM Tris-HCl, pH 8, 30% v/v glycerol, 2% w/v SDS, 22.5 mg/ml iodoacetamide) for 15 min on a rocking platform. Second dimension SDS-PAGE was performed by laying the strips on 12.5% isocratic Laemmli gels (24×20 cm), cast in low fluorescence glass plates, on an Ettan DALT Six system. Gels were run at 20°C at a constant power of 2.5 W per gel for 60 min followed by 17 W per gel until the bromophenol blue tracking front had run off the end of the gel. Triplicate gels were run for each sample using independent biological replicates. Fluorescence images of the gels were obtained on a Typhoon 9400 scanner (GE Healthcare). Cy2, Cy3 and Cy5 images were scanned at excitation/emission wavelengths of 488/520 nm, 532/580 nm and 633/670 nm, respectively, at a resolution of 100 µm. Both image analysis and statistical quantification of relative protein levels were performed using Progenesis SameSpots V.4 (Nonlinear Dynamics) (See Table S1 for detailed statistics of each spot). The data were analyzed as pairwise comparisons.

### Protein Identification by Mass Spectrometry

In order to excise the spots of interest, gels were silver stained as described elsewhere [50]. Protein spots of interest were excised from the gel using a cut tip. The selected spots are those that are differentially expressed in at least two clinical isolates. In-gel trypsin digestion was performed as described previously [51]. MALDI-MS analysis of tryptic peptides was performed on an Ultraflex time-of-flight instrument (Bruker Daltonics). Samples were prepared using α-cyano-4-hydroxy-cinnamic acid. Calibration was performed in the external mode using a peptide calibration standard kit (Bruker Daltonics). The spectra were processed using Flex Analysis 2.2 software (Bruker Daltonics). Peak lists were generated using the signals in the 800–4000 mass:charge ratio (*m/z*) range, with a signal:noise threshold >3. The SNAP algorithm included in the software was used to select the monoisotopic peaks from the isotopic distributions observed. After removing *m/z* values corresponding to commonly observed matrix cluster ions, an internal statistical calibration was applied. Peaks corresponding to frequently seen keratin and trypsin autolysis peptides were then removed. The resulting final peak list was used for the identification of the proteins by peptide mass fingerprint. The Mascot 2.0 program (Matrix Science) was used to search the NCBI non-redundant database (http://ncbi.nlm.nih.gov, March 2010), with no limitation on taxonomy. Search parameters were as follows: trypsin cleavages excluding N-terminal to P, one missed cleavage permission, carbamidomethylation and methionine oxidation as variable modification, mass tolerance <50 ppm, monoisotopic mass values. Criteria for positive identification were a significant Mascot probability score (*p*<0.05), and at least five matching peptide masses. A minimum score of 83, and a >50-point difference between this score and the score of the second ranked non-homologous match was obtained for all the identified differentially expressed proteins.

### Ethics Statement

*Stenotrophomonas maltophilia* strains were obtained from the internal collection of Hospital Municipal de Badalona (Barcelona, Spain) and have no link with patient data.

Zebrafish were handled in compliance with Directive 2010/63/EU of the European Parliament and of the Council on the protection of animals used for scientific purposes and with Decree 214/1997 of the Government of Catalonia, which regulates the use of animals for experimental and other scientific purposes. Experimental protocols have been reviewed and approved by the Animal and Human Experimentation Ethics Committee (CEEAH) of the Universitat Autònoma de Barcelona (UAB), Spain (ref # CEEAH-1968).

## Supporting Information

Figure S1 Growth curves of the four *S. maltophilia* strains in Luria-Bertani (LB) media at 37°C and 250 rpm.
(TIF)

Figure S2 Representative image of the gels obtained for M30 vs. ATCC13637. Superimposed images in pseudo-color from Cy3 (green, clinical isolate) and Cy5 (red, ATCC13637 strain) labeled samples run on a two-dimensional DIGE gel. The horizontal dimension corresponds to isoelectric point (*pI*) and ranges from 3 (left) to 10 (right). The vertical dimension corresponds to mass and ranges from ≈15 kDa (bottom) to ≈200 kDa (top).
(TIF)

Figure S3 DIGE image analysis from the M30/ATCC13637 comparison. (a) Superimposed images in pseudo-color from Cy3 (green, clinical isolate) and Cy5 (red, ATCC13637 strain) labeled samples run on the two-dimensional gel. The horizontal dimension corresponds to isoelectric point (*pI*) and ranges from 3 (left) to 10 (right). The vertical dimension corresponds to mass and ranges from ≈15 kDa (bottom) to ≈200 kDa (top). The six spots with largest difference in protein abundance between the two samples are marked. Three-dimensional images representing the intensity of these spots, corresponding to the Cy3 image (M30 strain, left-hand panel for each protein) and the Cy5 image (collection strain, right-hand panel for each protein), are shown. (b) Standardized abundance plots for the six proteins. Each graph displays the abundance observed for the spot in each of the three gel images corresponding to the clinical isolate sample (pink) and the ATCC13637 strain sample (blue), after standardizing the values using the internal standard pool images (Cy2) of each of the three gels. The line links the average abundance values for each group of samples. ANOVA’s test for the difference in abundance between each two groups results in *p* values <0.05 in all cases shown.
(TIF)

Table S1 *S. maltophilia* proteins presenting significant abundance difference in the clinical and ATCC13637 strains.
(DOCX)

Table S2 Statistical report for each identified DIGE spot.
(DOCX)

Table S3 Orthologs of the differentially abundant proteins reported to be involved in pathogenesis, virulence or resistance mechanisms.
(DOCX)
