# Supplementary figures and images for: Abundance of the Quorum-Sensing Factor Ax21 in Four Strains of Stenotrophomonas maltophilia Correlates with Mortality Rate in a New Zebrafish Model of Infection
Source: PLoS One. 2013 Jun 26;8(6):e67207. doi: 10.1371/journal.pone.0067207 (PMC3693955; doi:10.1371/journal.pone.0067207)

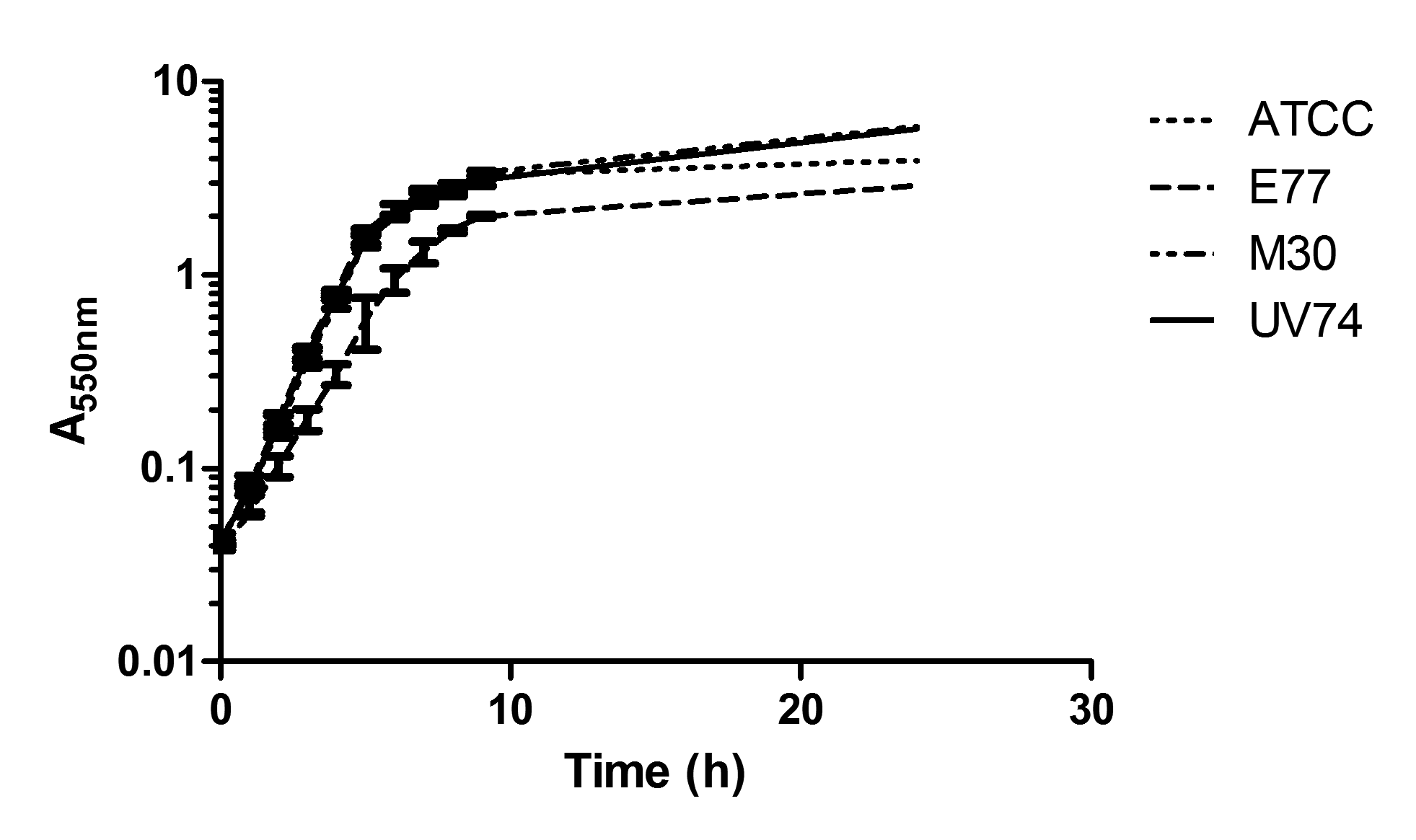

Supplement: Figure S1 — Growth curves of the four S. maltophilia strains in Luria-Bertani (LB) media at 37°C and 250 rpm. (TIF) [file pone.0067207.s001.tif]

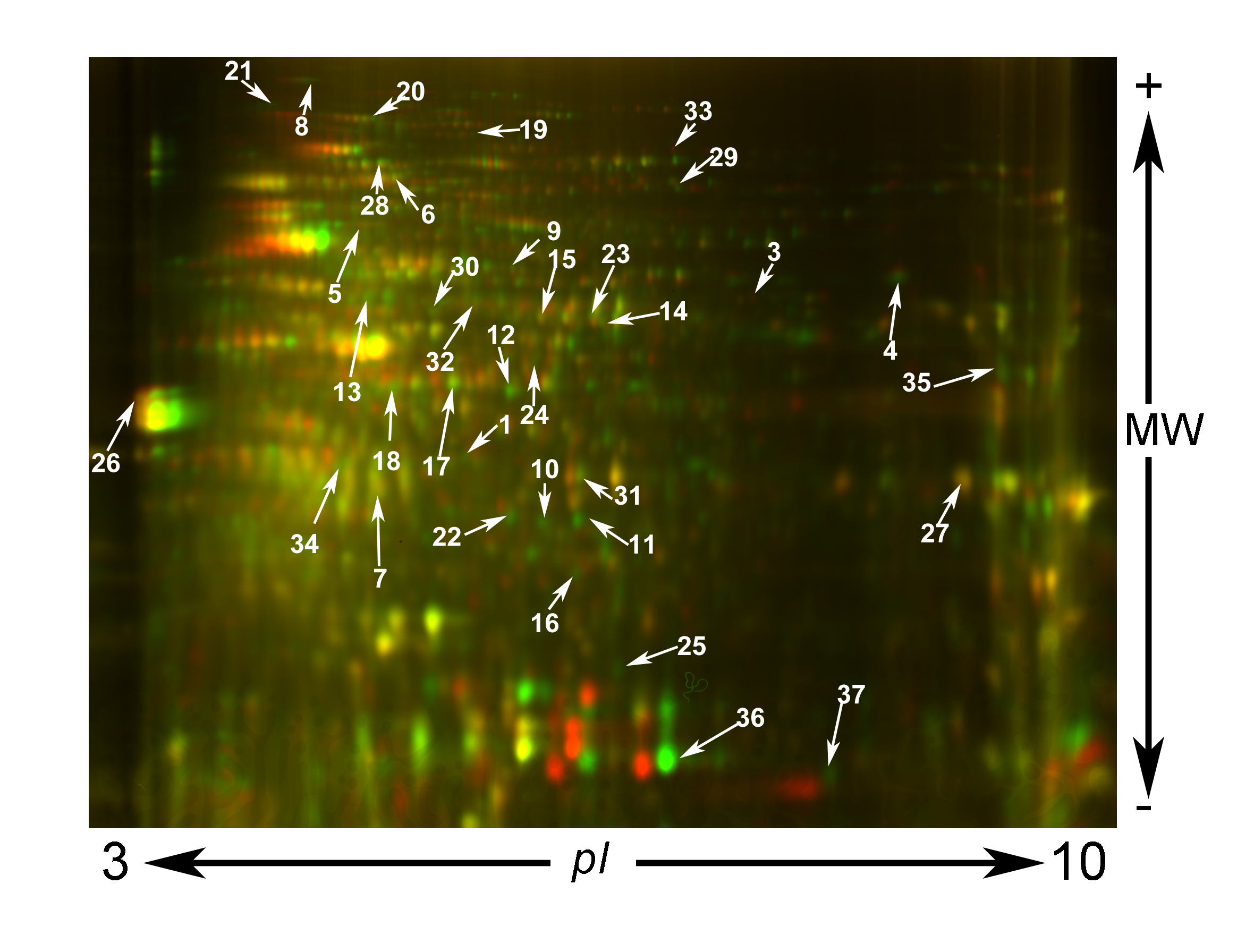

Supplement: Figure S2 — Representative image of the gels obtained for M30 vs. ATCC13637. Superimposed images in pseudo-color from Cy3 (green, clinical isolate) and Cy5 (red, ATCC13637 strain) labeled samples run on a two-dimensional DIGE gel. The horizontal dimension corresponds to isoelectric point (pI) and ranges from 3 (left) to 10 (right). The vertical dimension corresponds to mass and ranges from ≈15 kDa (bottom) to ≈200 kDa (top). (TIF) [file pone.0067207.s002.tif]

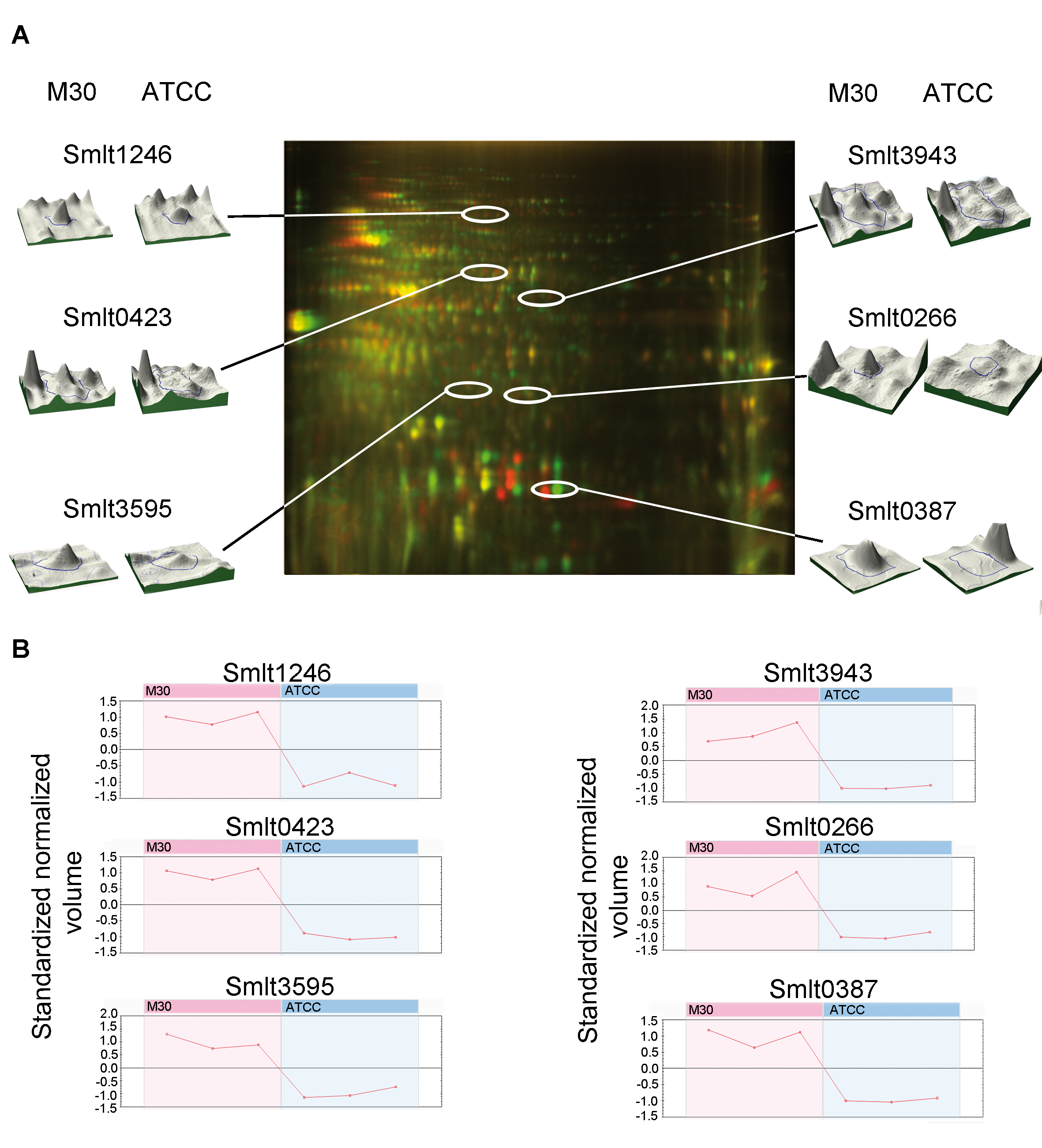

Supplement: Figure S3 — DIGE image analysis from the M30/ATCC13637 comparison. (a) Superimposed images in pseudo-color from Cy3 (green, clinical isolate) and Cy5 (red, ATCC13637 strain) labeled samples run on the two-dimensional gel. The horizontal dimension corresponds to isoelectric point (pI) and ranges from 3 (left) to 10 (right). The vertical dimension corresponds to mass and ranges from ≈15 kDa (bottom) to ≈200 kDa (top). The six spots with largest difference in protein abundance between the two samples are marked. Three-dimensional images representing the intensity of these spots, corresponding to the Cy3 image (M30 strain, left-hand panel for each protein) and the Cy5 image (collection strain, right-hand panel for each protein), are shown. (b) Standardized abundance plots for the six proteins. Each graph displays the abundance observed for the spot in each of the three gel images corresponding to the clinical isolate sample (pink) and the ATCC13637 strain sample (blue), after standardizing the values using the internal standard pool images (Cy2) of each of the three gels. The line links the average abundance values for each group of samples. ANOVA’s test for the difference in abundance between each two groups results in p values <0.05 in all cases shown. (TIF) [file pone.0067207.s003.tif]
