# Supplementary material for: Abundance of the Quorum-Sensing Factor Ax21 in Four Strains of Stenotrophomonas maltophilia Correlates with Mortality Rate in a New Zebrafish Model of Infection
Source: PLoS One. 2013 Jun 26;8(6):e67207. doi: 10.1371/journal.pone.0067207 (PMC3693955; doi:10.1371/journal.pone.0067207)
Supplement: Table S1 — S. maltophilia proteins presenting significant abundance difference in the clinical and ATCC13637 strains. (DOCX) [file pone.0067207.s004.docx]

**Table S1**. *S. maltophilia* proteins presenting significant abundance difference in the clinical and ATCC13637 strains.

| # | Protein name | Locus ID^a^ | Ratio^b^ M30 | Ratio^b^ UV74 | Ratio^b^ E77 | MASCOT Score^c^ | Peptides identified^c^ | Sequence coverage (%)^c^ | pI^d^ | MW (Da)^d^ | Biological process^h^ |
| --- | --- | --- | --- | --- | --- | --- | --- | --- | --- | --- | --- |
| 1 | Malonyl-CoA:acyl carrier protein transacylase (FabD) | Smlt1028 | 1.9 | 5.7 | 2.7 | 86 | 6 | 39.5% | 5.27 | 32494 | Fatty-acid biosynthesis |
| 2 | Acetyl-CoA carboxylase, carboxyl transferase alpha subunit (AccA) | Smlt1490 | N.D. | 2.1 | 1.1 | 114 | 10 | 36.4% | 8.91 | 35133 | Fatty-acid biosynthesis, metabolism of pyruvate, propanoate, terpenoids and polyketides |
| 3 | Acetyl-CoA carboxylase, biotin carboxylase subunit (AccC) | Smlt4241 | -1.3 | -4.8 | 1.7 | 99 | 10 | 35.8% | 6.55 | 49241 | Fatty-acid biosynthesis, pyruvate and propanoate metabolism |
| 4 | Acetyl-CoA synthetase (AcsA) | Smlt4623 | 2.5 | 3.3 | 3.9 | 161 | 19 | 39.3% | 5.55 | 71696 | Glycolysis/gluconeogenesis, metabolism of pyruvate, propanoate, methane |
| 5 | Pyruvate dehydrogenase, dihydrolipoamide acetyltransferase subunit (PdhB) | Smlt4121 | -1.6 | 4.2 | 2.1 | 113 | 18 | 31.2% | 5.50 | 58279 | Glycolysis/gluconeogenesis, TCA cycle, pyruvate metabolism |
| 6 | Acetyl-CoA acetyltransferase (FadI) | Smlt0164 | 1.7 | -3.5 | 1.6 | 132 | 13 | 39.9% | 7.15 | 45840 | Metabolism of lipids, amino acids, carbohydrates, terpenoids and polyketides and xenobiotics |
| 7 | 2-oxoglutarate dehydrogenase, dihydrolipoamide succinyltransferase subunit (SucB) | Smlt3198 | 1.2 | -3.5 | 1.7 | 109 | 9 | 29.3% | 5.71 | 42018 | TCA cycle, Lys degradation |
| 8 | Aconitate hydratase 2 (AcnB) | Smlt2245 | 1.7 | 5.8 | 3.9 | 270 | 30 | 40.3% | 5.27 | 92896 | TCA cycle, metabolism of propanoate, glyoxylate and dicarboxylate |
| 9 | Methylmalonate-semialdehyde dehydrogenase (DntE) | Smlt0264 | 1.5 | 2.3 | 2.1 | 174 | 16 | 40.5% | 5.61 | 53443 | Val, Leu, Ile degradation, propanoate metabolism, β-Ala metabolism |
| 10 | Enoyl-CoA hydratase | Smlt0266 | 2.9 | 3.5 | 3.8 | 104 | 7 | 53.2% | 5.91 | 28136 | Metabolism of fatty acids, Phe, Trp, β-Ala, propanoate, butanoate; degradation of Val, Leu, Ile, Lys, terpenoids, polyketides, xenobiotics |
| 11 | Electron transfer flavoprotein, beta subunit (EtfB) | Smlt0646 | 1.9 | 1.5 | 1.5 | 89 | 5 | 33.1% | 6.20 | 26764 | Electron carrier activity in nitrogen metabolism |
| 12 | Butyryl-CoA dehydrogenase (Bcd) | Smlt3174 | 3.2 | 1.3 | 2.1 | 133 | 10 | 34.6% | 5.57 | 41278 | Metabolism of fatty acids and butanoate, degradation of Val, Leu, Ile |
| 13 | UDP-N-acetylglucosamine 1-carboxyvinyl-transferase (MurA) | Smlt1119 | 2.6 | 2.1 | 2.3 | 142 | 17 | 48.0% | 5.67 | 44487 | Peptidoglycan biosynthesis, amino sugar and nucleotide sugar metabolism |
| 14 | Serine hydroxymethyl-transferase (GlyA) | Smlt0718 | 1.6 | 2.4 | 1.8 | 84 | 7 | 18.2% | 6.12 | 44979 | Metabolism of Gly, Ser, Thr, cyanoamino acid, methane cofactors and vitamines |
| 15 | Leucine aminopeptidase (PepA) | Smlt0675 | 1.2 | 1.6 | 1.6 | 213 | 19 | 48.9% | 5.35 | 51203 | Glutathione metabolism, protein processing and turnover |
| 16 | Uridylate kinase (PyrH) | Smlt1504 | 1.7 | 1.7 | N.D. | 98 | 9 | 38.4% | 5.56 | 26048 | Pyrimidine biosynthesis |
| 17 | DNA-directed RNA polymerase, alpha subunit (RpoA) | Smlt0931 | 3.2 | 2.1 | 1.4 | 100 | 7 | 32.2% | 5.59 | 36315 | Transcription, purine and pyrimidine metabolism |
| 18 | dTDP-glucose 4,6-dehydratase (RfbB) | Smlt0647 | 1.8 | 1.3 | 2.1 | 125 | 10 | 39.6% | 5.46 | 38281 | Polyketide sugar unit biosynthesis, LPS O-antigen biosynthesis |
| 19 | LPS-assembly protein LptD | Smlt0821 | 1.5 | -6.5 | 15.0 | 140 | 21 | 34.0% | 6.67 | 93925 | LPS transport across de outer membrane |
| 20 | Putative TonB-dependent receptor | Smlt3444 | 2.9 | 2.1 | 8.8 | 219 | 30 | 43.0% | 5.83 | 101968 | Substrate-specific outer-membrane transporter |
| 21 | Putative TonB-dependent receptor | Smlt4151 | 3.3 | 6.8 | 12.6 | 125 | 19 | 29.3% | 5.29 | 102762 | Substrate-specific outer-membrane transporter |
| 22 | Putative membrane protease, HflC subunit | Smlt3595 | 1.4 | 1.8 | 1.5 | 236 | 18 | 67.2% | 6.04 | 31902 | Regulation of ATP-dependent protease FtsH (HflB) |
| 23 | Putative long-chain fatty acid transport protein (FadL) | Smlt0423 | 1.5 | 2.0 | 2.6 | 154 | 13 | 45.1% | 6.00 | 49980 | Outer-membrane LCFA transport |
| 24 | Porin P (OprP) | Smlt3943 | 2.2 | 1.8 | 2.8 | 234 | 19 | 46.3% | 6.16 | 42941 | Phosphate-selective porin |
| 25 | Putative outer membrane protein W (OmpW) | Smlt4123 | 1.7 | 2.3 | 1.3 | 161 | 13 | 56.4% | 7.91 | 22595 | Outer-membrane receptor, porin |
| 26 | Putative outer membrane protein A (OmpA) | Smlt0955 | -2.6 | 6.9 | 4.4 | 220 | 13 | 51.4% | 4.80 | 39490 | Outer-membrane receptor, porin |
| 27 | Putative outer membrane protein A (OmpA) | Smlt1826 | 1.4 | 2.2 | N.D. | 149 | 11 | 59.3% | 9.33 | 24521 | Outer-membrane receptor, porin |
| 28 | Endopeptidase O (PepO) | Smlt3447 | 2.2 | 4.2 | 3.9 | 113 | 12 | 29.0% | 5.62 | 80083 | Protein post-translational processing and turnover |
| 29 | Putative exported peptidase S9 | Smlt1246 | 1.8 | 3.9 | 3.6 | 150 | 20 | 38.0% | 6.13 | 77515 | Serine-type peptidase |
| 30 | Xaa-Pro dipeptidase (PepQ) | Smlt3861 | 1.6 | 2.6 | 1.7 | 132 | 12 | 30.5% | 5.61 | 48678 | Peptide catabolic processes |
| 31 | L-Threonine 3-dehydrogenase (Tdh) | Smlt0961 | 1.5 | 1.5 | 1.6 | 95 | 10 | 40.3% | 6.21 | 37013 | Glycine, serine and threonine metabolism |
| 32 | Adenylosuccinate lyase (PurB) | Smlt3193 | 1.8 | 1.7 | 2.1 | 160 | 14 | 40.7% | 5.68 | 49795 | Metabolism of purines, Ala, Asp, Glu |
| 33 | NADH-quinone oxidoreductase, G subunit (NuoG) | Smlt3399 | -1.7 | -3.2 | 4.0 | 170 | 22 | 40.1% | 6.56 | 78770 | Oxidative phosphorylation, nitrogen metabolism |
| 34 | Bifunctional molybdenum cofactor biosynthesis protein (MoaC) | Smlt2781 | 1.5 | 1.4 | 2.8 | 115 | 20 | 58.6% | 6.67 | 33986 | Molybdenum cofactor biosynthesis |
| 35 | Putative monooxygenase | Smlt1459 | 1.6 | 2.3 | 1.7 | 105 | 13 | 39.0% | 9.84 | 42337 | Ubiquinone biosynthesis |
| 36 | Putative quorum-sensing factor Ax21 | Smlt0387 | 9.2 | 11.0 | 3.4 | 112 | 10 | 50.0% | 6.06 | 21033 | Quorum-sensing signal molecule |
| 37 | UPF0234 family protein | Smlt4090 | 1.2 | 2.0 | 4.1 | 112 | 8 | 50.6% | 6.62 | 18000 | Uncharacterized |
| 38 | Conserved hypothetical exported protein | Smlt3796 | N.D. | 8.8 | 1.2 | 220 | 13 | 51.4% | 4.68 | 39357 | Uncharacterized |

^a^Locus ID in *Stenotrophomonas maltophilia* K279a, GenBank code AM743169.

^b^Abundance ratio clinical strain:ATCC13637; negative signs are indicative for the inverse ratio; N.D. stands for not detected in the clinical strain.

^c^Example statistics (from best identification).

^d^Theoretical isoelectric point and molecular weight.

^f^Biological process inferred from orthology relations or domain homologies as annotated in Uniprot (http://www.uniprot.org/), KEGG (www.genome.jp/kegg), InterPro (www.ebi.ac.uk/interpro) and references therein.
