# Supplementary material for: Abundance of the Quorum-Sensing Factor Ax21 in Four Strains of Stenotrophomonas maltophilia Correlates with Mortality Rate in a New Zebrafish Model of Infection
Source: PLoS One. 2013 Jun 26;8(6):e67207. doi: 10.1371/journal.pone.0067207 (PMC3693955; doi:10.1371/journal.pone.0067207)
Supplement: Table S2 — Statistical report for each identified DIGE spot. (DOCX) [file pone.0067207.s005.docx]

**Table S2**. Statistical report for each identified DIGE spot.

| Spot | Locus ID^a^ | Replica | Normalized volume | | | | | |
| --- | --- | --- | --- | --- | --- | --- | --- | --- |
|  |  |  | M30 | ATCC | UV74 | ATCC | E77 | ATCC |
| 1 | Smlt1028 | Replica 1 | 1.667 | 0.909 | 2.017 | 0.366 | 1.815 | 0.737 |
|  |  | Replica 2 | 1.560 | 0.896 | 1.688 | 0.258 | 2.116 | 0.857 |
|  |  | Replica 3 | 1.936 | 0.949 | 1.340 | 0.267 | 2.370 | 0.704 |
|  |  | Anova (*p*) | 0.0007 | | 0.0004 | | 0.0005 | |
| 2 | Smlt1490 | Replica 1 | N.D. | N.D. | 2.301 | 1.070 | 1.101 | 0.924 |
|  |  | Replica 2 | N.D. | N.D. | 2.113 | 1.116 | 1.258 | 0.878 |
|  |  | Replica 3 | N.D. | N.D. | 2.430 | 1.075 | 1.179 | 0.884 |
|  |  | Anova (*p*) | N.D. | | 7·10^-5^ | | 0.0027 | |
| 3 | Smlt4241 | Replica 1 | 0.811 | 1.321 | 1.358 | 0.308 | 1.103 | 0.574 |
|  |  | Replica 2 | 0.943 | 1.617 | 1.319 | 0.303 | 1.297 | 0.625 |
|  |  | Replica 3 | 0.924 | 1.378 | 1.441 | 0.363 | 1.167 | 0.524 |
|  |  | Anova (*p*) | 0.0036 | | 2·10^-5^ | | 0.0005 | |
| 4 | Smlt4623 | Replica 1 | 1.410 | 0.451 | 1.818 | 0.503 | 1.297 | 0.279 |
|  |  | Replica 2 | 1.235 | 0.440 | 1.774 | 0.494 | 1.355 | 0.408 |
|  |  | Replica 3 | 1.361 | 0.314 | 1.422 | 0.543 | 1.268 | 0.323 |
|  |  | Anova (*p*) | 0.0006 | | 0.0001 | | 0.0003 | |
| 5 | Smlt4121 | Replica 1 | 0.811 | 1.321 | 1.358 | 0.308 | 1.103 | 0.574 |
|  |  | Replica 2 | 0.943 | 1.617 | 1.319 | 0.303 | 1.297 | 0.625 |
|  |  | Replica 3 | 0.924 | 1.378 | 1.441 | 0.363 | 1.167 | 0.524 |
|  |  | Anova (*p*) | 0.0036 | | 2·10^-5^ | | 0.0005 | |
| 6 | Smlt0164 | Replica 1 | 1.369 | 0.820 | 0.330 | 1.379 | 1.030 | 0.644 |
|  |  | Replica 2 | 1.497 | 0.860 | 0.466 | 1.145 | 1.119 | 0.726 |
|  |  | Replica 3 | 1.505 | 0.878 | 0.324 | 1.415 | 0.999 | 0.658 |
|  |  | Anova (*p*) | 0.0001 | | 0.0007 | | 0.0009 | |
| 7 | Smlt3198 | Replica 1 | 0.868 | 1.171 | 0.633 | 1.290 | 1.573 | 0.669 |
|  |  | Replica 2 | 0.890 | 1.174 | 0.516 | 1.365 | 1.728 | 0.813 |
|  |  | Replica 3 | 0.940 | 1.160 | 0.530 | 1.380 | 1.705 | 0.752 |
|  |  | Anova (*p*) | 0.0004 | | 0.0002 | | 0.0002 | |
| 8 | Smlt2245 | Replica 1 | 1.249 | 0.856 | 1.910 | 0.211 | 1.407 | 0.119 |
|  |  | Replica 2 | 1.441 | 1.057 | 1.897 | 0.327 | 1.583 | 0.811 |
|  |  | Replica 3 | 1.653 | 0.709 | 1.466 | 0.378 | 1.481 | 0.202 |
|  |  | Anova (*p*) | 0.0222 | | 0.0008 | | 0.0407 | |
| 9 | Smlt0264 | Replica 1 | 1.517 | 0.959 | 1..502 | 0.740 | 1.235 | 0.465 |
|  |  | Replica 2 | 1.556 | 1.057 | 1.441 | 0.633 | 1.353 | 0.537 |
|  |  | Replica 3 | 1.698 | 1.053 | 1.344 | 0.643 | 1.208 | 0.455 |
|  |  | Anova (*p*) | 0.0007 | | 0.0002 | | 0.0001 | |
| 10 | Smlt0266 | Replica 1 | 1.989 | 0.647 | 1.679 | 0.510 | 2.035 | 0.518 |
|  |  | Replica 2 | 1.547 | 0.567 | 1.492 | 0.474 | 2.630 | 0.681 |
|  |  | Replica 3 | 1.946 | 0.690 | 1.449 | 0.323 | 2.049 | 0.577 |
|  |  | Anova (*p*) | 0.0004 | | 0.0010 | | 0.0003 | |
| 11 | Smlt0646 | Replica 1 | 1.849 | 1.012 | 1.206 | 0.808 | 1.190 | 0.824 |
|  |  | Replica 2 | 1.722 | 0.943 | 1.102 | 0.689 | 1.350 | 0.891 |
|  |  | Replica 3 | 2.046 | 1.014 | 0.980 | 0.755 | 1.339 | 0.836 |
|  |  | Anova (*p*) | 0.0003 | | 0.0075 | | 0.0009 | |
| 12 | Smlt3174 | Replica 1 | 1.769 | 0.558 | 0.929 | 0.731 | 1.329 | 0.545 |
|  |  | Replica 2 | 1.656 | 0.612 | 0.909 | 0.726 | 1.118 | 0.685 |
|  |  | Replica 3 | 1.929 | 0.512 | 0.930 | 0.681 | 1.312 | 0.591 |
|  |  | Anova (*p*) | 7·10^-5^ | | 0.0004 | | 0.0001 | |
| 13 | Smlt1119 | Replica 1 | 2.076 | 0.725 | 2.495 | 1.166 | 1.271 | 0.537 |
|  |  | Replica 2 | 2.075 | 0.667 | 2.624 | 1.132 | 1.258 | 0.487 |
|  |  | Replica 3 | 1.798 | 0.932 | 2.038 | 1.047 | 1.398 | 0.653 |
|  |  | Anova (*p*) | 0.0010 | | 0.0008 | | 0.0007 | |
| 14 | Smlt0718 | Replica 1 | 1.494 | 0.933 | 1.309 | 0.567 | 1.073 | 0.686 |
|  |  | Replica 2 | 1.549 | 1.067 | 1.082 | 0.404 | 1.158 | 0.747 |
|  |  | Replica 3 | 1.644 | 1.017 | 1.228 | 0.532 | 1.113 | 0.427 |
|  |  | Anova (*p*) | 0.0008 | | 0.0017 | | 0.0248 | |
| 15 | Smlt0675 | Replica 1 | 1.197 | 1.066 | 0.973 | 0.630 | 1.169 | 0.647 |
|  |  | Replica 2 | 1.338 | 0.929 | 1.027 | 0.665 | 1.203 | 0.848 |
|  |  | Replica 3 | 1.220 | 1.087 | 0.959 | 0.532 | 1.147 | 0.699 |
|  |  | Anova (*p*) | 0.0298 | | 0.0023 | | 0.0044 | |
| 16 | Smlt1504 | Replica 1 | 1.472 | 0.863 | 1.044 | 0.710 | N.D. | N.D. |
|  |  | Replica 2 | 1.291 | 0.778 | 1.120 | 0.623 | N.D. | N.D. |
|  |  | Replica 3 | 1.386 | 0.836 | 1.148 | 0.626 | N.D. | N.D. |
|  |  | Anova (*p*) | 0.0004 | | 0.0005 | | N.D. | |
| 17 | Smlt0931 | Replica 1 | 1.769 | 0.558 | 1.084 | 0.530 | 1.063 | 0.820 |
|  |  | Replica 2 | 1.656 | 0.612 | 1.132 | 0.552 | 1.135 | 0.993 |
|  |  | Replica 3 | 1.929 | 0.512 | 1.058 | 0.485 | 1.092 | 0.799 |
|  |  | Anova (*p*) | 7·10^-5^ | | 7·10^-5^ | | 0.0295 | |
| 18 | Smlt0647 | Replica 1 | 1.415 | 0.558 | 0.929 | 0.731 | 1.329 | 0.545 |
|  |  | Replica 2 | 1.519 | 0.612 | 0.909 | 0.726 | 1.118 | 0.685 |
|  |  | Replica 3 | 1.512 | 0.512 | 0.930 | 0.681 | 1.312 | 0.591 |
|  |  | Anova (*p*) | 0.0011 | | 0.0011 | | 0.0005 | |
| 19 | Smlt0821 | Replica 1 | 1.688 | 1.090 | 0.203 | 1.244 | 1.397 | 0.091 |
|  |  | Replica 2 | 2.003 | 1.344 | 0.181 | 1.292 | 1.474 | 0.149 |
|  |  | Replica 3 | 2.036 | 1.278 | 0.213 | 1.369 | 1.393 | 0.042 |
|  |  | Anova (*p*) | 0.0075 | | 4·10^-6^ | | 0.0015 | |
| 20 | Smlt3444 | Replica 1 | 1.853 | 0.589 | 1.288 | 0.623 | 1.509 | 0.106 |
|  |  | Replica 2 | 2.303 | 0.787 | 1.195 | 0.564 | 1.331 | 0.221 |
|  |  | Replica 3 | 1.860 | 0.686 | 1.225 | 0.573 | 1.350 | 0.148 |
|  |  | Anova (*p*) | 0.0006 | | 4·10^-5^ | | 0.0005 | |
| 21 | Smlt4151 | Replica 1 | 1.884 | 0.410 | 1.577 | 0.373 | 1.340 | 0.159 |
|  |  | Replica 2 | 1.430 | 0.495 | 1.025 | 0.583 | 1.411 | 0.287 |
|  |  | Replica 3 | 1.555 | 0.556 | 1.206 | 0.492 | 1.330 | 0.154 |
|  |  | Anova (*p*) | 0.0006 | | 0.0059 | | 0.0006 | |
| 22 | Smlt3595 | Replica 1 | 1.624 | 1.074 | 1.200 | 0.808 | 1.332 | 0.835 |
|  |  | Replica 2 | 1.451 | 0.868 | 1.195 | 0.674 | 1.234 | 0.903 |
|  |  | Replica 3 | 1.583 | 1.013 | 1.314 | 0.547 | 1.319 | 0.823 |
|  |  | Anova (*p*) | 0.0003 | | 0.0063 | | 0.0004 | |
| 23 | Smlt0423 | Replica 1 | 1.318 | 0.901 | 1.209 | 0.653 | 1.309 | 0.445 |
|  |  | Replica 2 | 1.238 | 0.830 | 1.216 | 0.612 | 1.249 | 0.621 |
|  |  | Replica 3 | 1.239 | 0.848 | 1.227 | 0.522 | 1.279 | 0.464 |
|  |  | Anova (*p*) | 0.0003 | | 0.0004 | | 0.0009 | |
| 24 | Smlt3943 | Replica 1 | 1.636 | 0.704 | 1.423 | 0.817 | 1.227 | 0.485 |
|  |  | Replica 2 | 1.735 | 0.765 | 1.315 | 0.741 | 1.298 | 0.422 |
|  |  | Replica 3 | 1.822 | 0.855 | 1.324 | 0.674 | 1.221 | 0.447 |
|  |  | Anova (*p*) | 0.0002 | | 0.0006 | | 2·10^-5^ | |
| 25 | Smlt4123 | Replica 1 | 1.487 | 0.864 | 1.551 | 0.582 | 1.287 | 0.647 |
|  |  | Replica 2 | 1.5 | 0.849 | 1.343 | 0.594 | 1.205 | 0.590 |
|  |  | Replica 3 | 1.425 | 0.852 | 1.196 | 0.568 | 1.238 | 0.524 |
|  |  | Anova (*p*) | 5·10^-6^ | | 0.0004 | | 0.0003 | |
| 26 | Smlt0955 | Replica 1 | 0.577 | 1.310 | 2.006 | 0.198 | 1.365 | 0.214 |
|  |  | Replica 2 | 0.479 | 1.552 | 1.111 | 0.301 | 1.309 | 0.246 |
|  |  | Replica 3 | 0.565 | 1.311 | 1.737 | 0.201 | 1.381 | 0.454 |
|  |  | Anova (*p*) | 0.0003 | | 0.0010 | | 0.0026 | |
| 27 | Smlt1826 | Replica 1 | 1.229 | 0.847 | 2.158 | 1.218 | N.D. | N.D. |
|  |  | Replica 2 | 1.131 | 0.836 | 2.314 | 1.050 | N.D. | N.D. |
|  |  | Replica 3 | 1.277 | 0.927 | 2.440 | 0.860 | N.D. | N.D. |
|  |  | Anova (*p*) | 0.0023 | | 0.0017 | | N.D. | |
| 28 | Smlt3447 | Replica 1 | 1.446 | 0.625 | 1.358 | 0.308 | 1.297 | 0.279 |
|  |  | Replica 2 | 1.374 | 0.735 | 1.319 | 0.303 | 1.355 | 0.408 |
|  |  | Replica 3 | 1.552 | 0.598 | 1.441 | 0.363 | 1.268 | 0.328 |
|  |  | Anova (*p*) | 0.0004 | | 2·10^-5^ | | 0.0003 | |
| 29 | Smlt1246 | Replica 1 | 1.281 | 0.747 | 2.083 | 0.638 | 1.348 | 0.302 |
|  |  | Replica 2 | 1.131 | 0.694 | 1.981 | 0.370 | 1.240 | 0.430 |
|  |  | Replica 3 | 1.375 | 0.713 | 1.484 | 0.424 | 1.160 | 0.297 |
|  |  | Anova (*p*) | 0.0008 | | 0.0022 | | 0.0005 | |
| 30 | Smlt3861 | Replica 1 | 1.482 | 0.995 | 1.840 | 0.548 | 1.160 | 0.565 |
|  |  | Replica 2 | 1.566 | 0.995 | 1.371 | 0.628 | 1.174 | 0.711 |
|  |  | Replica 3 | 1.458 | 0.884 | 1.275 | 0.573 | 1.119 | 0.768 |
|  |  | Anova (*p*) | 0.0005 | | 0.0014 | | 0.0046 | |
| 31 | Smlt0961 | Replica 1 | 1.550 | 0.978 | 1.473 | 0.784 | 1.042 | 0.692 |
|  |  | Replica 2 | 1.554 | 1.074 | 1.024 | 0.781 | 1.138 | 0.771 |
|  |  | Replica 3 | 1.648 | 1.057 | 1.023 | 0.828 | 1.127 | 0.591 |
|  |  | Anova (*p*) | 0.0003 | | 0.0391 | | 0.0042 | |
| 32 | Smlt3193 | Replica 1 | 1.260 | 0.754 | 1.188 | 0.706 | 1.379 | 0.484 |
|  |  | Replica 2 | 1.259 | 0.698 | 1.148 | 0.674 | 1.495 | 0.841 |
|  |  | Replica 3 | 1.254 | 0.684 | 1.208 | 0.684 | 1.266 | 0.627 |
|  |  | Anova (*p*) | 4·10^-5^ | | 1·10^-5^ | | 0.0097 | |
| 33 | Smlt3399 | Replica 1 | 0.886 | 1378 | 0.392 | 1.257 | 1.327 | 0.249 |
|  |  | Replica 2 | 0.956 | 1.674 | 0.430 | 1.273 | 1.423 | 0.437 |
|  |  | Replica 3 | 0.856 | 1.539 | 0.391 | 1.365 | 1.365 | 0.351 |
|  |  | Anova (*p*) | 0.0012 | | 9·10^-6^ | | 0.0010 | |
| 34 | Smlt2781 | Replica 1 | 1.355 | 0.855 | 0.997 | 0.757 | 1.337 | 0.283 |
|  |  | Replica 2 | 1.271 | 0.880 | 1.204 | 0.693 | 1.265 | 0.533 |
|  |  | Replica 3 | 1.377 | 0.902 | 0.964 | 0.738 | 1.253 | 0.542 |
|  |  | Anova (*p*) | 0.0001 | | 0.0079 | | 0.0071 | |
| 35 | Smlt1459 | Replica 1 | 1.785 | 1.198 | 1.817 | 0.678 | 1.426 | 0.747 |
|  |  | Replica 2 | 1.861 | 1.070 | 1.376 | 0.728 | 1.169 | 0.895 |
|  |  | Replica 3 | 2.027 | 1.186 | 1.864 | 0.811 | 1.498 | 0.731 |
|  |  | Anova (*p*) | 0.0007 | | 0.0018 | | 0.0054 | |
| 36 | Smlt0387 | Replica 1 | 2.415 | 0.236 | 2.233 | 0.193 | 1.320 | 0.365 |
|  |  | Replica 2 | 1.863 | 0.204 | 1.821 | 0.165 | 1.267 | 0.387 |
|  |  | Replica 3 | 2.386 | 0.285 | 1.484 | 0.145 | 1.275 | 0.388 |
|  |  | Anova (*p*) | 7·10^-5^ | | 8·10^-5^ | | 1·10^-6^ | |
| 37 | Smlt4090 | Replica 1 | 1.005 | 0.894 | 2.333 | 0.881 | 1.430 | 0.326 |
|  |  | Replica 2 | 1.149 | 0.894 | 1.904 | 1.203 | 1.441 | 0.604 |
|  |  | Replica 3 | 1.042 | 0.821 | 1.456 | 0.728 | 1.443 | 0.125 |
|  |  | Anova (*p*) | 0.0147 | | 0.0239 | | 0.0252 | |
| 38 | Smlt3796 | Replica 1 | N.D. | N.D. | 1.919 | 0.233 | 1.005 | 0.904 |
|  |  | Replica 2 | N.D. | N.D. | 1.908 | 0.247 | 1.107 | 0.812 |
|  |  | Replica 3 | N.D. | N.D. | 1.794 | 0.156 | 1.133 | 0.940 |
|  |  | Anova (*p*) | N.D. | | 0.0001 | | 0.025 | |

^a^Locus ID in *Stenotrophomonas maltophilia* K279a, GenBank code AM743169.

N.D.: Not Detected
