# Supplementary material for: Abundance of the Quorum-Sensing Factor Ax21 in Four Strains of Stenotrophomonas maltophilia Correlates with Mortality Rate in a New Zebrafish Model of Infection
Source: PLoS One. 2013 Jun 26;8(6):e67207. doi: 10.1371/journal.pone.0067207 (PMC3693955; doi:10.1371/journal.pone.0067207)
Supplement: Table S3 — Orthologs of the differentially abundant proteins reported to be involved in pathogenesis, virulence or resistance mechanisms. (DOCX) [file pone.0067207.s006.docx]

**Table S3**. Orthologs of the differentially abundant proteins reported to be involved in pathogenesis, virulence or resistance mechanisms.

| # | Protein name | Locus ID^a^ | Orthologs in pathogenesis/virulence/resistance | Interactions^b^ |
| --- | --- | --- | --- | --- |
| 1 | Malonyl-CoA:acyl carrier protein transacylase (FabD) | Smlt1028 | Involved in biosynthesis of coronatine (*Pseudomonas syringae*) [1]  Involved in resistance to trifluoroperazine (*Escherichia coli*) [2] | AccA AccC |
| 2 | Acetyl-CoA carboxylase, carboxyl transferase alpha subunit (AccA) | Smlt1490 |  | AccC FabD AcsA PdhB FadI |
| 3 | Acetyl-CoA carboxylase, biotin carboxylase subunit (AccC) | Smlt4241 | Downregulated by epinephrin (*Salmonella enterica*) [3] | AccA FabD AcsA PdhB FadI |
| 4 | Acetyl-CoA synthetase (AcsA) | Smlt4623 | Essential for growth on ethanol (*Pseudomonas aeruginosa*) [4,5] | AccC DntE FadI PdhB AccA |
| 5 | Pyruvate dehydrogenase, dihydrolipoamide acetyltransferase subunit (PdhB) | Smlt4121 | Involved in host interactions (*Mycoplasma pneumoniae*) [6]  Belongs to core exoproteome of virulent strains (*Staphylococcus aureus*) [7]  PDH complex required for pathogenesis (*Mycobacterium tuberculosis*) [8]  Immunoreactive (*Bacillus* sp.) [9]  Involved in type III secretion system-dependent cytotoxicity (*Pseudomonas aeruginosa*) [10] | AcsA AccC AccA FadI |
| 6 | Acetyl-CoA acetyltransferase (FadI) | Smlt0164 |  | AcsA AccA PdhB AccA SucB |
| 7 | 2-oxoglutarate dehydrogenase, dihydrolipoamide succinyltransferase subunit (SucB) | Smlt3198 | Involved in persister survival and tolerance to antibiotics and stress (*Escherichia coli*) [11]  Immunoreactive (*Bartonella henselae*) [12] | FadI AcnB |
| 8 | Aconitate hydratase 2 (AcnB) | Smlt2245 | Involved in colonization (*Xanthomonas campestris*) [13]  Involved in virulence (*Xanthomonas oryzae*) [14]  Upregulated under oxidative stress together with virulence factors / involved in resistance to hydrogen peroxyde [15,16,17] | SucB |
| 9 | Methylmalonate-semialdehyde dehydrogenase (DntE) | Smlt0264 |  | AcsA Smlt0266 |
| 10 | Enoyl-CoA hydratase | Smlt0266 | Involved in virulence (*Pseudomonas aeruginosa*) [18] | DntE EtfB |
| 11 | Electron transfer flavoprotein, beta subunit (EtfB) | Smlt0646 |  | Bcd Smlt0266 |
| 12 | Butyryl-CoA dehydrogenase (Bcd) | Smlt3174 |  | EtfB |
| 13 | UDP-N-acetylglucosamine 1-carboxyvinyl-transferase (MurA) | Smlt1119 | Upregulation confers resistance to fosfomycin and to bromoacetate (*Escherichia coli*) [19,20]  Required for intrinsic resistance to cephalosporin (*Enterococcus faecalis*) [21]  Upregulated in biofilm (*Streptococcus suis*) [22] | GlyA |
| 14 | Serine hydroxymethyl-transferase (GlyA) | Smlt0718 | Upregulated by the QS system (*Pseudomonas aeruginosa*) [23] | PepA MurA PyrH |
| 15 | Leucine aminopeptidase (PepA) | Smlt0675 | Highly upregulated by the QS system together with virulence factors (*Pseudomonas aeruginosa*) [23,24] | GlyA |
| 16 | Uridylate kinase (PyrH) | Smlt1504 | Involved in biofilm, QS and virulence (*Pseudomonas aeruginosa*, *Vibrio vulnificus*) [25,26]  Essential for in-host survival (*Vibrio vulnificus*) [27] | RpoA GlyA |
| 17 | DNA-directed RNA polymerase, alpha subunit (RpoA) | Smlt0931 | Involved in the activation of virulence factors (*Salmonella enterica*, *Brucella melitensis*) [28,29] | PyrH |
| 18 | dTDP-glucose 4,6-dehydratase (RfbB) | Smlt0647 | Involved in colonization and virulence (*Salmonella* Typhimurium) [30]  Involved in colonization (*Vibrio cholerae*) [31]  Upregulated by the QS system (*Pseudomonas aeruginosa*) [23] |  |
| 19 | LPS-assembly protein LptD | Smlt0821 | Δ mutants are leaky and produce little LPS, which is not in the surface (*Neisseria meningitidis*) [32] |  |
| 20  21 | Putative TonB-dependent receptor | Smlt3444  Smlt4151 | Involved in virulence (*Pseudomonas fluorescens*, *Neisseria meningitidis*, *Flavobacterium psychrophilum*, *Porphyromonas gingivalis*, *Escherichia coli*, *Bordetella avium*) [33,34,35,36,37,38] |  |
| 22 | Putative membrane protease, HflC subunit | Smlt3595 | Involved in tobramycin resistance (*Pseudomonas aeruginosa*) [39] |  |
| 23 | Putative long-chain fatty acid transport protein (FadL) | Smlt0423 | Involved in virulence (*Pseudomonas fluorescens*) [40]  Involved in chlortetracycline resistance (down regulation in presence of CTC, but increased sensitivity of Δ mutant) (*Escherichia coli*) [41]  Upregulated in response to erythromycin, kanamycin, tetracycline, streptomycin, and chloromycetin (*Vibrio alginolyticus*) [42] |  |
| 24 | Porin P (OprP) | Smlt3943 |  |  |
| 25 | Putative outer membrane protein W (OmpW) | Smlt4123 | Involved in virulence [43]  Involved in resistance to tetracycline, ceftriaxone and oxidative stress (by downregulation) (*Acinetobacter baumannii*, *Salmonella* Typhimurium, *Escherichia coli*) [44,45,46]  Involved in resistance to paraquat (by upregulation) (*Salmonella* Typhimurium) [47] |  |
| 26  27 | Putative outer membrane protein A (OmpA) | Smlt0955  Smlt1826 | Involved in virulence (*Acinetobacter baumannii*, *Francisella tularensis*) [48,49]  Involved in adhesion/invasion (*Escherichia coli*, *Porphyromonas gingivalis*) [50,51,52] |  |
| 28 | Endopeptidase O (PepO) | Smlt3447 | Involved in virulence (*Aeromonas hydrophila*) [53]  Involved in invasion/colonization (*Porphyromonas gingivalis*) [54] |  |
| 29 | Putative exported peptidase S9 | Smlt1246 |  |  |
| 30 | Xaa-Pro dipeptidase (PepQ) | Smlt3861 |  |  |
| 31 | L-Threonine 3-dehydrogenase (Tdh) | Smlt0961 |  |  |
| 32 | Adenylosuccinate lyase (PurB) | Smlt3193 | Involved i virulence (*Salmonella enterica*) [55]  Involved in colonization (*Listeria monocytogenes*) [56]  Involved in biofilm formation and virulence (*Streptococcus sanguinis*) [57] |  |
| 33 | NADH-quinone oxidoreductase, G subunit (NuoG) | Smlt3399 | Inhibits apoptosis of infected host cells (*Mycobacterium tuberculosis*) [58,59,60]  Involved in virulence (*Salmonella* Gallinarum) [61] |  |
| 34 | Bifunctional molybdenum cofactor biosynthesis protein (MoaC) | Smlt2781 |  |  |
| 35 | Putative monooxygenase | Smlt1459 |  |  |
| 36 | Putative quorum-sensing factor Ax21 | Smlt0387 | Involved in regulation of motility, biofilm formation and virulence (*Xanthomonas oryzae*) [62,63]  Involved in intraspecies signaling (non sulfation-dependent) (*Stenotrophomonas maltophilia*) [64] |  |
| 37 | UPF0234 family protein | Smlt4090 |  |  |
| 38 | Conserved hypothetical exported protein | Smlt3796 |  |  |

^a^Locus ID in *Stenotrophomonas maltophilia* K279a, GenBank code AM743169.

^b^Interaction partners within the list of differentially abundant proteins as given by STRING (<http://string-db.org/>), based on *Stenotrophomonas maltophilia* K279a using all prediction methods and a confidence threshold of 0.7.

**REFERENCES**

1. Seidle H, Rangaswamy V, Couch R, Bender CL, Parry RJ (2004) Characterization of Cfa1, a monofunctional acyl carrier protein involved in the biosynthesis of the phytotoxin coronatine. J Bacteriol 186: 2499-2503.

2. Bouquin N, Tempête M, Holland IB, Séror SJ (1995) Resistance to trifluoroperazine, a calmodulin inhibitor, maps to the fabD locus in *Escherichia coli*. Mol Gen Genet 246: 628-637.

3. Spencer H, Karavolos MH, Bulmer DM, Aldridge P, Chhabra SR et al. (2010) Genome-wide transposon mutagenesis identifies a role for host neuroendocrine stress hormones in regulating the expression of virulence genes in *Salmonella*. J Bacteriol 192: 714-724.

4. Kretzschmar U, Khodaverdi V, Adrian L (2010) Transcriptional regulation of the acetyl-CoA synthetase gene acsA in *Pseudomonas aeruginosa*. Arch Microbiol 192:685-690.

5. Kretzschmar U, Schobert M, Görisch H (2001) The *Pseudomonas aeruginosa* acsA gene, encoding an acetyl-CoA synthetase, is essential

6. Thomas C, Jacobs E, Dumke R (2012) Characterization of pyruvate dehydrogenase subunit B and enolase as plasminogen binding proteins in *Mycoplasma pneumoniae*. Microbiology [Epub ahead of print].

7. Wolf C, Kusch H, Monecke S, Albrecht D, Holtfreter S et al. (2011) Genomic and proteomic characterization of *Staphylococcus aureus* mastitis isolates of bovine origin. Proteomics 11: 2491-2502.

8. Venugopal A, Bryk R, Shi S, Rhee K, Rath P et al. (2011) Virulence of *Mycobacterium tuberculosis* depends on lipoamide dehydrogenase, a member of three multienzyme complexes. Cell Host Microbe 9: 21-31.

9. Delvecchio VG, Connolly JP, Alefantis TG, Walz A, Quan MA et al. (2006) Proteomic profiling and identification of immunodominant spore antigens of *Bacillus anthracis*, *Bacillus cereus*, and *Bacillus thuringiensis*. Appl Environ Microbiol 72: 6355-6363.

10. Dacheux D, Epaulard O, de Groot A, Guery B, Leberre R et al. (2002) Activation of the *Pseudomonas aeruginosa* type III secretion system requires an intact pyruvate dehydrogenase aceAB operon. Infect Immun 70: 3973-3977.

11. Ma C, Sim S, Shi W, Du L, Xing D et al. (2010) Energy production genes sucB and ubiF are involved in persister survival and tolerance to multiple antibiotics and stresses in *Escherichia coli*. FEMS Microbiol Lett 303: 33-40.

12. Litwin CM, Johnson JM, Martins TB (2004) The *Bartonella henselae* sucB gene encodes a dihydrolipoamide succinyltransferase protein reactive with sera from patients with cat-scratch disease. J Med Microbiol 53: 1221-1227.

13. Kirchberg J, Büttner D, Thiemer B, Sawers RG (2012) Aconitase B is required for optimal growth of *Xanthomonas campestris* pv. *vesicatoria* in pepper plants. PLoS One 7: e34941.

14. Subramoni S, Pandey A, Vishnu Priya MR, Patel HK, Sonti RV (2012) The ColRS system of *Xanthomonas oryzae* pv. *oryzae* is required for virulence and growth in iron-limiting conditions. Mol Plant Pathol 13: 690-703.

15. Huang CH, Chiou SH (2011) Proteomic analysis of upregulated proteins in *Helicobacter pylori* under oxidative stress induced by hydrogen peroxide. Kaohsiung J Med Sci 27: 544-553.

16. Tang Y, Quail MA, Artymiuk PJ, Guest JR, Green J (2002) *Escherichia coli* aconitases and oxidative stress: post-transcriptional regulation of sodA expression. Microbiology 148: 1027-1037.

17. Kim JN, Kwon YM (2013) Genetic and phenotypic characterization of the RyhB regulon in *Salmonella Typhimurium*. Microbiol Res 168: 41-49.

18. Feinbaum RL, Urbach JM, Liberati NT, Djonovic S, Adonizio A et al. (2012) Genome-wide identification of *Pseudomonas aeruginosa* virulence-related genes using a *Caenorhabditis elegans* infection model. PLoS Pathog 8: e1002813.

19. Couce A, Briales A, Rodríguez-Rojas A, Costas C, Pascual A et al. (2012) Genomewide overexpression screen for fosfomycin resistance in *Escherichia coli*: MurA confers clinical resistance at low fitness cost. Antimicrob Agents Chemother 56: 2767-2769.

20. Desai KK, Miller BG (2010) Recruitment of genes and enzymes conferring resistance to the nonnatural toxin bromoacetate. Proc Natl Acad Sci U S A 107: 17968-17973.

21. Vesić D, Kristich CJ (2012) MurAA is required for intrinsic cephalosporin resistance of *Enterococcus faecalis*. Antimicrob Agents Chemother 56: 2443-2451.

22. Wang Y, Yi L, Wu Z, Shao J, Liu G et al. (2012) Comparative proteomic analysis of *Streptococcus suis* biofilms and planktonic cells that identified biofilm infection-related immunogenic proteins. PLoS One 7: e33371.

23. Schuster M, Lostroh CP, Ogi T, Greenberg EP (2993) Identification, timing, and signal specificity of *Pseudomonas aeruginosa* quorum-controlled genes: a transcriptome analysis. J Bacteriol 185: 2066-2079.

24. Sarnovsky R, Rea J, Makowski M, Hertle R, Kelly C et al. (2009) Proteolytic cleavage of a C-terminal prosequence, leading to autoprocessing at the N Terminus, activates leucine aminopeptidase from *Pseudomonas aeruginosa*. J Biol Chem 284: 10243-10253.

25. Naughton S, Parker D, Seemann T, Thomas T, Turnbull L et al. (2011) *Pseudomonas aeruginosa* AES-1 exhibits increased virulence gene expression during chronic infection of cystic fibrosis lung. PLoS One 6: e24526.

26. Shin NR, Lee DY, Yoo HS (2007) Identification of quorum sensing-related regulons in *Vibrio vulnificus* by two-dimensional gel electrophoresis and differentially displayed reverse transcriptase PCR. FEMS Immunol Med Microbiol 50: 94-103.

27. Lee SE, Kim SY, Kim CM, Kim MK, Kim YR et al. (2007) The pyrH gene of *Vibrio vulnificus* is an essential in vivo survival factor. Infect Immun 75: 2795-2801.

28. Olekhnovich IN, Kadner RJ (2004) Contribution of the RpoA C-terminal domain to stimulation of the *Salmonella enterica* hilA promoter by HilC and HilD. J Bacteriol 186: 3249-3253.

29. Lestrate P, Dricot A, Delrue RM, Lambert C, Martinelli V et al. (2003) Attenuated signature-tagged mutagenesis mutants of *Brucella melitensis* identified during the acute phase of infection in mice. Infect Immun 71: 7053-7060.

30. Turner AK, Lovell MA, Hulme SD, Zhang-Barber L, Barrow PA (1998) Identification of *Salmonella* typhimurium genes required for colonization of the chicken alimentary tract and for virulence in newly hatched chicks. Infect Immun 66: 2099-2106.

31. Chiang SL, Mekalanos JJ (1999) rfb mutations in *Vibrio cholerae* do not affect surface production of toxin-coregulated pili but still inhibit intestinal colonization. Infect Immun 67: 976-980.

32. Bos MP, Tommassen J (2011) The LptD chaperone LptE is not directly involved in lipopolysaccharide transport in *Neisseria meningitidis*. J Biol Chem 286: 28688-28696.

33. Hu YH, Dang W, Sun L (2012) A TonB-dependent outer membrane receptor of *Pseudomonas fluorescens*: virulence and vaccine potential. Arch Microbiol 194: 795-802.

34. Stork M, Bos MP, Jongerius I, de Kok N, Schilders I et al. (2010) An outer membrane receptor of *Neisseria meningitidis* involved in zinc acquisition with vaccine potential. PLoS Pathog 6: e1000969.

35. Alvarez B, Alvarez J, Menendez A, Guijarro JA (2008) A mutant in one of two exbD loci of a TonB system in *Flavobacterium psychrophilum* shows attenuated virulence and confers protection against cold water disease. Microbiology 154: 1144-1151.

36. Shi X, Hanley SA, Faray-Kele MC, Fawell SC et al. (2007) The rag locus of *Porphyromonas gingivalis* contributes to virulence in a murine model of soft tissue destruction. Infect Immun 75: 2071-2074.

37. Torres AG, Redford P, Welch RA, Payne SM (2001) TonB-dependent systems of uropathogenic *Escherichia coli*: aerobactin and heme transport and TonB are required for virulence in the mouse. Infect Immun 69: 6179-6185.

38. Murphy ER, Sacco RE, Dickenson A, Metzger DJ, Hu Y et al. (2002) BhuR, a virulence-associated outer membrane protein of *Bordetella avium*, is required for the acquisition of iron from heme and hemoproteins. Infect Immun 70: 5390-5403.

39. Hinz A, Lee S, Jacoby K, Manoil C. Membrane proteases and aminoglycoside antibiotic resistance. J Bacteriol 2011, 193: 4790-4797.

40. de Lima Pimenta A, Di Martino P, Le Bouder E, Hulen C, Blight MA (2003) *In vitro* identification of two adherence factors required for *in vivo* virulence of *Pseudomonas fluorescens*. Microbes Infect 5: 1177-1187.

41. Lin XM, Yang JN, Peng XX, Li H (2010) A novel negative regulation mechanism of bacterial outer membrane proteins in response to antibiotic resistance. J Proteome Res 9: 5952-5959.

42. Xiong XP, Wang C, Ye MZ, Yang TC, Peng XX et al. (2010) Differentially expressed outer membrane proteins of *Vibrio alginolyticus* in response to six types of antibiotics. Mar Biotechnol (NY) 12: 686-695

43. McClean S (2012) Eight Stranded β-Barrel and Related Outer Membrane Proteins: Role in Bacterial Pathogenesis. Protein Pept Lett 19: 1013-1025.

44. Yun SH, Choi CW, Park SH, Lee JC, Leem SH et al. (2008) Proteomic analysis of outer membrane proteins from *Acinetobacter baumannii* DU202 in tetracycline stress condition. J Microbiol 46: 720-727.

45. Hu WS, Li PC, Cheng CY (2005) Correlation between ceftriaxone resistance of *Salmonella enterica* serovar Typhimurium and expression of outer membrane proteins OmpW and Ail/OmpX-like protein, which are regulated by BaeR of a two-component system. Antimicrob Agents Chemother 49: 3955-3958.

46. Asakura H, Kawamoto K, Haishima Y, Igimi S, Yamamoto S et al. (2008) Differential expression of the outer membrane protein W (OmpW) stress response in enterohemorrhagic *Escherichia coli* O157:H7 corresponds to the viable but non-culturable state. Res Microbiol 159: 709-717.

47. Gil F, Ipinza F, Fuentes J, Fumeron R, Villarreal JM et al. (2007) The ompW (porin) gene mediates methyl viologen (paraquat) efflux in *Salmonella enterica* serovar typhimurium. Res Microbiol 158: 529-536.

48. McConnell MJ, Actis L, Pachón J (2012) *Acinetobacter baumannii*: human infections, factors contributing to pathogenesis and animal models. FEMS Microbiol Rev doi: 10.1111/j.1574-6976.2012.00344.x

49. Mahawar M, Atianand MK, Dotson RJ, Mora V, Rabadi SM et al. (2012) Identification of a novel *Francisella tularensis* factor required for intramacrophage survival and subversion of innate immune response. J Biol Chem 287: 25216-25229.

50. Xie Y, Kim KJ, Kim KS (2004) Current concepts on *Escherichia coli* K1 translocation of the blood-brain barrier. FEMS Immunol Med Microbiol 42: 271-279.

51. Selvaraj SK, Periandythevar P, Prasadarao NV (2007) Outer membrane protein A of *Escherichia coli* K1 selectively enhances the expression of intercellular adhesion molecule-1 in brain microvascular endothelial cells. Microbes Infect 9: 547-557.

52. Komatsu T, Nagano K, Sugiura S, Hagiwara M, Tanigawa N (2012) E-selectin mediates *Porphyromonas gingivalis* adherence to human endothelial cells. Infect Immun 80: 2570-2576.

53. Abolghait SK, Akeda Y, Kodama T, Cantarelli VV, Iida T et al. (2010) *Aeromonas hydrophila* PepO outer membrane endopeptidase activates human big endothelin-3 in vitro and induces skin ulcer in goldfish (Carassius auratus). Vet Microbiol 145: 113-121.

54. Ansai T, Yu W, Urnowey S, Barik S, Takehara T (2003) Construction of a pepO gene-deficient mutant of *Porphyromonas gingivalis*: potential role of endopeptidase O in the invasion of host cells. Oral Microbiol Immunol 18: 398-400.

55. McFarland WC, Stocker BA (1987) Effect of different purine auxotrophic mutations on mouse-virulence of a Vi-positive strain of *Salmonella* Dublin and of two strains of *Salmonella* Typhimurium. Microb Pathog 3: 129-141.

56. Faith NG, Kim JW, Azizoglu R, Kathariou S, Czuprynski C (2012) Purine biosynthesis mutants (purA and purB) of serotype 4b *Listeria monocytogenes* are severely attenuated for systemic infection in intragastrically inoculated A/J Mice. Foodborne Pathog Dis 9: 480-486.

57. Ge X, Kitten T, Chen Z, Lee SP, Munro CL et al. (2008) Identification of *Streptococcus sanguinis* genes required for biofilm formation and examination of their role in endocarditis virulence. Infect Immun 76: 2551-2559.

58. Blomgran R, Desvignes L, Briken V, Ernst JD (2012) *Mycobacterium tuberculosis* inhibits neutrophil apoptosis, leading to delayed activation of naive CD4 T cells. Cell Host Microbe 11: 81-90.

59. Miller JL, Velmurugan K, Cowan MJ, Briken V (2010) The type I NADH dehydrogenase of *Mycobacterium tuberculosis* counters phagosomal NOX2 activity to inhibit TNF-alpha-mediated host cell apoptosis. PLoS Pathog 6: e1000864.

60. Velmurugan K, Chen B, Miller JL, Azogue S, Gurses S et al. (2007) *Mycobacterium tuberculosis* nuoG is a virulence gene that inhibits apoptosis of infected host cells. PLoS Pathog 3: e110.

61. Zhang-Barber L, Turner AK, Dougan G, Barrow PA (1998) Protection of chickens against experimental fowl typhoid using a nuoG mutant of *Salmonella* serotype Gallinarum. Vaccine 16: 899-903.

62. Ham JH (2012) Intercellular and intracellular signalling systems that globally control the expression of virulence genes in plant pathogenic bacteria. Mol Plant Pathol doi: 10.1111/mpp.12005

63. Ronald PC (2011) Small protein-mediated quorum sensing in a gram-negative bacterium: novel targets for control of infectious disease. Discov Med 12: 461-470.

64. McCarthy Y, Dow JM, Ryan RP (2011) The Ax21 protein is a cell-cell signal that regulates virulence in the nosocomial pathogen *Stenotrophomonas maltophilia*. J Bacteriol 193: 6375-6378.
